# Picornavirus 2A protease regulates stress granule formation to facilitate viral translation

**DOI:** 10.1371/journal.ppat.1006901

**Published:** 2018-02-07

**Authors:** Xiaodan Yang, Zhulong Hu, Shanshan Fan, Qiang Zhang, Yi Zhong, Dong Guo, Yali Qin, Mingzhou Chen

**Affiliations:** State Key Laboratory of Virology and Modern Virology Research Center, College of Life Sciences, Wuhan University, LuoJia Hill, Wuhan, China; Stanford University, UNITED STATES

## Abstract

Stress granules (SGs) contain stalled messenger ribonucleoprotein complexes and are related to the regulation of mRNA translation. Picornavirus infection can interfere with the formation of SGs. However, the detailed molecular mechanisms and functions of picornavirus-mediated regulation of SG formation are not clear. Here, we found that the 2A protease of a picornavirus, EV71, induced atypical stress granule (aSG), but not typical stress granule (tSG), formation via cleavage of eIF4GI. Furthermore, 2A was required and sufficient to inhibit tSGs induced by EV71 infection, sodium arsenite, or heat shock. Infection of 2A protease activity-inactivated recombinant EV71 (EV71-2A^C110S^) failed to induce aSG formation and only induced tSG formation, which is PKR and eIF2α phosphorylation-dependent. By using a Renilla luciferase mRNA reporter system and RNA fluorescence in situ hybridization assay, we found that EV71-induced aSGs were beneficial to viral translation through sequestering only cellular mRNAs, but not viral mRNAs. In addition, we found that the 2A protease of other picornaviruses such as poliovirus and coxsackievirus also induced aSG formation and blocked tSG formation. Taken together, our results demonstrate that, on one hand, EV71 infection induces tSG formation via the PKR-eIF2α pathway, and on the other hand, 2A, but not 3C, blocks tSG formation. Instead, 2A induces aSG formation by cleaving eIF4GI to sequester cellular mRNA but release viral mRNA, thereby facilitating viral translation.

## Author summary

When cellular translation initiation is stalled, translation initiation complexes aggregate in cytoplasm. We call these aggregations stress granules (SGs), and they can be marked by components such as TIA-1. SGs are always considered to be antiviral structures during viral infection, but viruses also regulate SG formation to facilitate their survival. Here, we show that the 2A protease of EV71 induced TIA-1 foci formation, and we analyzed these TIA-1 foci and found that they were different from typical stress granules (tSGs); thus, we named them atypical stress granules (aSGs). 2A alone could block tSG formation, and we found that protease activity of 2A was required for aSG induction and tSG blockage, but functioned in different ways. When the protease activity of 2A in EV71 was blocked (EV71-2A^C110S^), the tSGs but not aSGs appeared in infected cells. These tSGs contained cellular and viral mRNAs and translation initiation factors to inhibit viral translation, but aSGs contained only cellular mRNAs to promote viral translation. We propose a model revealing that EV71 escapes cellular antiviral response by manipulating SG formation: 2A transforms the overall translation shutdown system to a selective virally beneficial system by switching from tSGs to aSGs.

## Introduction

Stress granules (SGs) form in response to a variety of stresses such as oxidative stress, heat shock (HS), hypoxia, nutrient deprivation, and viral infection [1]. During SG formation, messenger RNA (mRNA) translation initiation is inhibited, and polysomes are disassembled. Thus, SGs contain stalled pre-initiation complexes (PICs) consisting of translationally silent mRNAs, 40S ribosomal subunits, canonical eukaryotic initiation factors (eIFs) such as eIF4E, eIF4G, eIF4A, eIF4B, and eIF3, and RNA-binding proteins (RBPs). Two aggregation-prone RBPs, T-cell-restricted intracellular antigen 1 (TIA-1) and the RasGAP SH3-domain binding protein 1 (G3BP), appear to be critical for SG formation and are recruited to SGs [2,3].

SGs in general are considered to be transient and dynamic. Compounds such as cycloheximide (CHX) stabilize mRNAs on polysomes and inhibit SG formation and foster their disassembly [4,5]. The SG formation induced by the phosphorylation of eIF2α is well characterized. The protein kinase R (PKR), PKR-like ER kinase (PERK), general control nonderepressible 2 (GCN2), or heme regulated inhibitor (HRI) can phosphorylate eIF2α under different stress conditions. For example, PKR can be activated by viral dsRNA, and HRI can be activated by arsenite (AS) or HS. The phosphorylation of eIF2α interferes with the formation of the eIF2-GTP-tRNA_i_^Met^ ternary complex and thereby stalls translation initiation [1,6–8]. However, eIF2α-independent SG formation also exists and includes eIF4A inhibition by either pateamine A or hippuristanol and inhibition of eIF4G-eIF4E interactions during hydrogen peroxide-induced oxidative stress [9–12].

Thus, SG formation can be caused by a variety of mechanisms that impair translation initiation. Although SGs formed in response to diverse stresses share many of the same components, certain factors appear to be recruited in a stress-specific fashion. For example, HS protein 27 (HSP27) is found in SGs in HS cells but not in cells undergoing oxidative stress [13]; the p68 src-associated protein in mitosis (Sam68) is recruited to SGs in picornavirus-infected cells but not to those formed in response to oxidative stress or HS. Thus, SGs may be compositionally different depending on the type of stress [14], and distinct SGs may be regulated differentially and have multiple roles. However, the mechanisms by which SGs form have not been identified completely.

Enterovirus 71 (EV71), a member of the *Picornaviridae* family, is widely spread and causes severe hand-foot-mouth disease in infants [15]. Thus, understanding the host factors that influence viral pathogenesis is critical to designing improved antiviral strategies. The EV71 genome (∼7.5 kb) can be immediately translated into a single polyprotein via an internal ribosome entry sequence (IRES)-mediated, cap-independent mechanism of translation initiation, and this polyprotein is subsequently processed by proteases 2A and 3C into the structural and nonstructural proteins [16]. Furthermore, the IRES can drive the viral genome translation in the absence of functional eIF4F complex, which is disrupted due to cleavage of eIF4G in picornavirus-infected cells [17,18].

SGs are thought to be antiviral, and many viruses have hence evolved various strategies to disrupt SG formation to maintain efficient translation of their proteins and to prevent their genomes and transcripts from being stalled in SGs [19,20]. Poliovirus (PV) infection was initially indicated by the recruitment of HuR and G3BP to SGs in early phases [10]. Subsequently, White et al. found that eIF4G and polyA-binding protein (PABP) were also recruited to SGs early in PV infection [21]. However, further examination revealed that eIF4G, G3BP, and PABP were no longer found in SGs at later times, indicating that PV may actively disrupt SG formation at later times [21]. Furthermore, the discovery that G3BP was cleaved by 3C, which coincided with SG disassembly in infected cells, provided a possible explanation for these findings [21]. White et al. suggested a model whereby PV initially induces SG formation but induces SG disassembly at later stages via cleavage of G3BP, thus preventing SG formation even in the presence of external stress [21].

However, because most of the SG markers used in the study by White et al. can be cleaved by 2A or 3C, whether other SG components are also released from SGs is unclear. Therefore, Piotrowska et al. further examined SG formation in PV-infected cells and found that infection induced stable, compositionally unique SGs containing TIA-1 but lacking G3BP and eIF4G and that these SGs did not disassemble at late times in infected cells, which raised the possibility that PV might not induce the complete disassembly of SGs. As G3BP, eIF4G, and PABP are all cleaved by viral proteases, proteolysis may trigger their release from SGs. Alternatively, the release may be triggered by a mechanism not yet identified [14]. Furthermore, recent studies showed that 2A of picornaviruses induced SG formation and that no other viral proteins induced SG formation [22]. However, the molecular mechanism of these 2A-induced SGs and the cellular components that 2A targets to trigger SG formation remain unknown.

Therefore, several questions raised in the aforementioned studies have not been resolved. (1) How do picornaviruses induce SG formation at early times and block SG assembly at later times? (2) Is cleavage of G3BP by 3C critical for the inhibition of SG formation at later times during infection? (3) What is the molecular mechanism of 2A-induced SG formation? Are they typical stress granules (tSGs)? (4) What are the organization and role of 2A-induced SGs in picornavirus-infected cells?

In this study, we demonstrate that the 2A protease of EV71 blocks tSG formation but induces atypical stress granule (aSG) formation to facilitate viral translation. These aSGs are induced by cleavage of eIF4GI and are different from tSGs in that they are devoid of G3BP and a series of eIFs, they are independent of eIF2α and PKR phosphorylation, they cannot be dissolved by CHX, and they can specifically sequester cellular mRNAs but not viral mRNAs. On the other hand, infection with a 2A protease activity-inactivated recombinant virus, EV71-2A^C110S^, induces tSG formation via the PKR-eIF2α pathway, and these tSGs are antiviral structures. These findings provide a new conceptual mechanism for SG regulation during picornavirus infection.

## Results

### 2A induces aSG formation during EV71 infection

**Fig 1:**
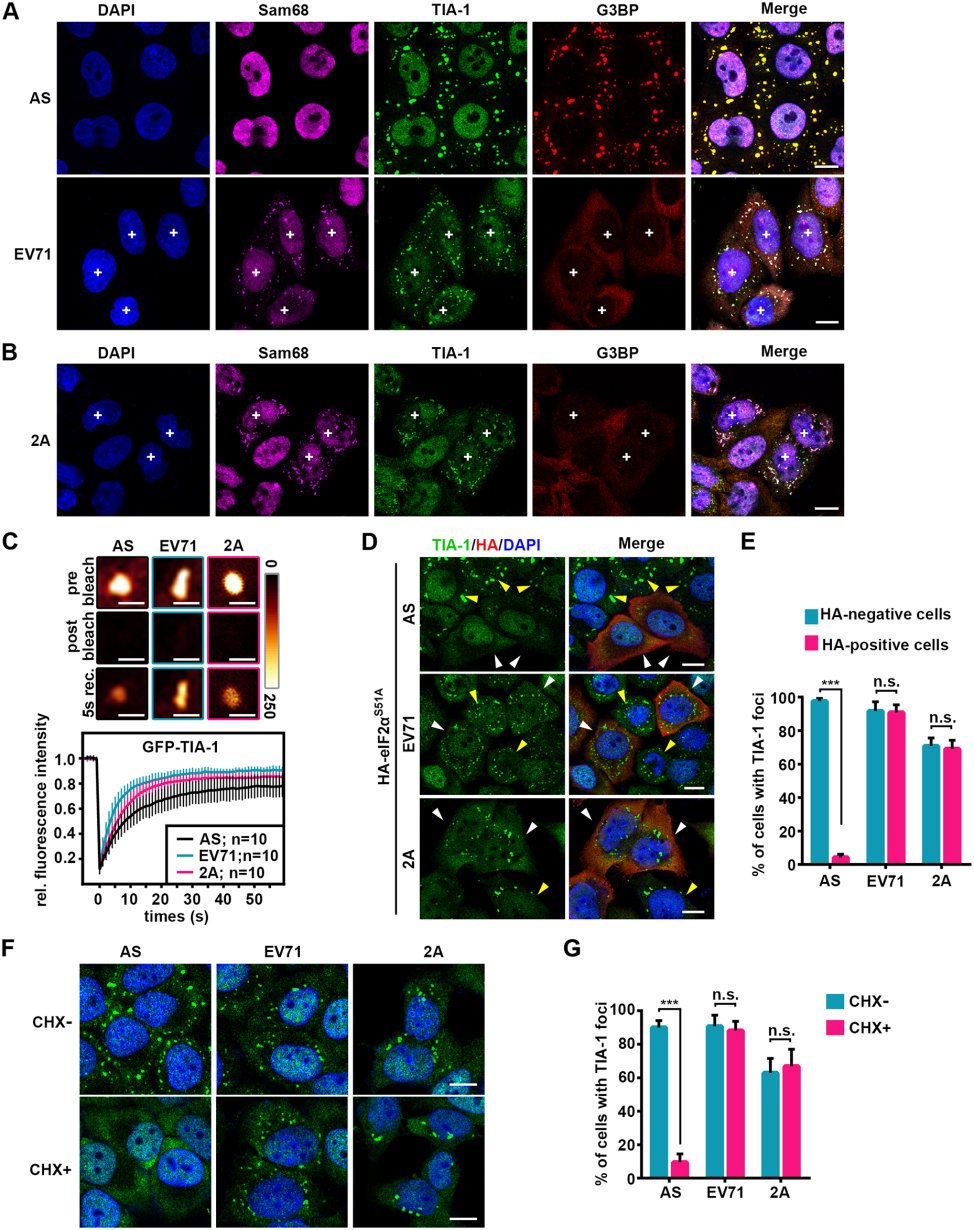
2A-induced stable TIA-1 foci are aSGs. (A-B) The location of Sam68, TIA-1, and G3BP in AS-treated (200 μM, 1 h), EV71-infected (MOI = 10, 5 hpi) (A) or 2A-expressing (24 hpt) (B) HeLa cells. “+” indicates EV71-infected or 2A-expressing cells. (C) Dynamics of TIA-1 in AS-, EV71-, or 2A-induced foci. FRAP assays were used to evaluate the averaged recovery of GFP-TIA-1 in the indicated foci. Representative images of fluorescence intensities observed in foci at indicated times during FRAP assays are shown in pseudo-colors (glowdark) in the top panels. (D) Effects of eIF2α phosphorylation on TIA-1 foci. Following transfection with HA-eIF2α^S51A^ for 24 h, HeLa cells were treated with AS, infected with EV71 for 5 h, or transfected with 2A for 24 h and then fixed and stained as indicated. White and yellow arrows show the comparison of TIA-1 foci assembly in the presence and absence of eIF2α^S51A^. (E) Quantitative analysis of the cells (in D) with TIA-1 foci among HA-positive or HA-negative cells. n = 3, 240 cells/condition were counted, mean±SD; n.s., no statistical significance, ***p<0.001. (F) CHX effects on TIA-1 foci. HeLa cells were treated with AS (0.5 h), EV71 (5 h), or 2A (24 h), with or without CHX for 1 h (CHX+ or CHX-) and then fixed and stained with TIA-1(green) and DAPI (blue). (G) Quantitative analysis of cells with TIA-1 foci in F. n = 3, 240 cells/condition were counted, mean±SD; n.s., no statistical significance, ***p<0.001. Scale bars in B, 2 μm; scale bars in others, 10 μm. See also S1 and S2 Figs.

To explore the formation of SGs during picornavirus infection, we infected HeLa cells with EV71 for 6 hours (h) and visualized SGs via immunofluorescence (IF) with antibodies against Sam68, TIA-1, and G3BP. EV71 infection led to the formation of SGs containing Sam68 and TIA-1, but devoid of G3BP, and as a control, AS induced the formation of tSGs containing TIA-1 and G3BP, but not Sam68 (Fig 1A).

To explore EV71-induced SG formation in more detail, we tried to clarify the dynamics of SG assembly during EV71 infection and visualized SGs at different time points post-infection (pi). An antibody against EV71 was used to visualize EV71-infected cells, and TIA-1 and Sam68 were also visualized. When cells were mock- or EV71-infected for 2 hours, EV71 infection was undetectable via IF, and localization of TIA-1 and Sam68 did not change. As infection proceeded, IF revealed EV71 in 90% of the cells at 4 hpi and 95% of the cells at 6 hpi, TIA-1 and Sam68 assembled into foci and colocalized with each other in infected cells (S1A and S1H Fig). Therefore, the appearance of TIA-1 and Sam68 foci can be used as indicators of EV71 infection. However, although TIA-1-related protein (TIAR) aggregated and persisted in infected cells with TIA-1 and Sam68, the other tSG markers such as G3BP, PABP, 40S ribosomal protein S3 (RPS3), eIF1a, eIF3a, eIF4G, eIF4A, and eIF4E aggregated in less than 30% of cells at 4 hpi and were evenly distributed at 6 hpi (S1B–S1H Fig). Similar results were observed in EV71-infected rhabdomyosarcoma (RD) cells (S1H Fig), suggesting that the persistent SGs containing TIA-1, TIAR, and Sam68 might not be tSGs.

To confirm which protein of EV71 induced the formation of persistent SGs, we expressed all the viral proteins in HeLa and RD cells and found that only 2A protease induced the formation of TIA-1 foci (S2A Fig), which also contained Sam68 and TIAR but did not contain PABP, G3BP, RPS3, eIF1a, eIF3a, eIF4G, eIF4A, or eIF4E (Fig 1B and S2B Fig), suggesting that 2A expression alone is sufficient to trigger the formation of persistent SGs during EV71 infection and the formation of Sam68 or TIA-1 foci can also be used as an indicator of 2A expression.

Next, we sought to determine whether 2A-induced persistent SGs share features with tSGs. First, a previous study showed that tSGs are dynamic structures of which TIA-1 rapidly shuttles in and out [23]. Thus, we analyzed the dynamics of TIA-1 in tSGs and persistent SGs via fluorescence recovery after photobleaching (FRAP) assay. To rule out the impact of size, typical and persistent SGs with similar size (1.5–2 μm in dimeter) were analyzed. We found that GFP-TIA-1 in typical and persistent SGs were rapidly recovered (Fig 1C), suggesting that persistent SGs are also dynamic structures. Second, previous studies also showed that stresses not only induce tSG formation but also induce adjacent processing body (p-body) formation [24–26]. Thus we also analyzed the location of the p-bodies in EV71-infected cells by using a widely used marker of p-bodies—mRNA-decapping enzyme 1A (DCP1A), and found that p-bodies were adjacent to EV71-induced persistent SGs at 4h post-infection, but disappeared at 6h post-infection, suggesting that EV71-induced persistent SGs were distinct from p-bodies but similar to tSGs (S2C and S2D Fig). Third, we evaluated the effect of eIF2α phosphorylation on the formation of TIA-1 foci and found that expression of eIF2α^S51A^ (an eIF2α non-phosphorylated mutant) [27–29] blocked AS-induced tSG formation but had no effect on EV71- and 2A-induced formation of persistent SGs (Fig 1D and 1E), indicating that 2A induced the formation of persistent SGs in a phospho-eIF2α-independent manner. Fourth, we found that CHX dispersed AS-induced tSGs but had no effect on EV71- and 2A-induced persistent SGs (Fig 1F and 1G). Therefore, we defined EV71- and 2A-induced persistent SGs as atypical stress granules (aSGs).

### 2A induces aSGs by cleaving eIF4GI

**Fig 2:**
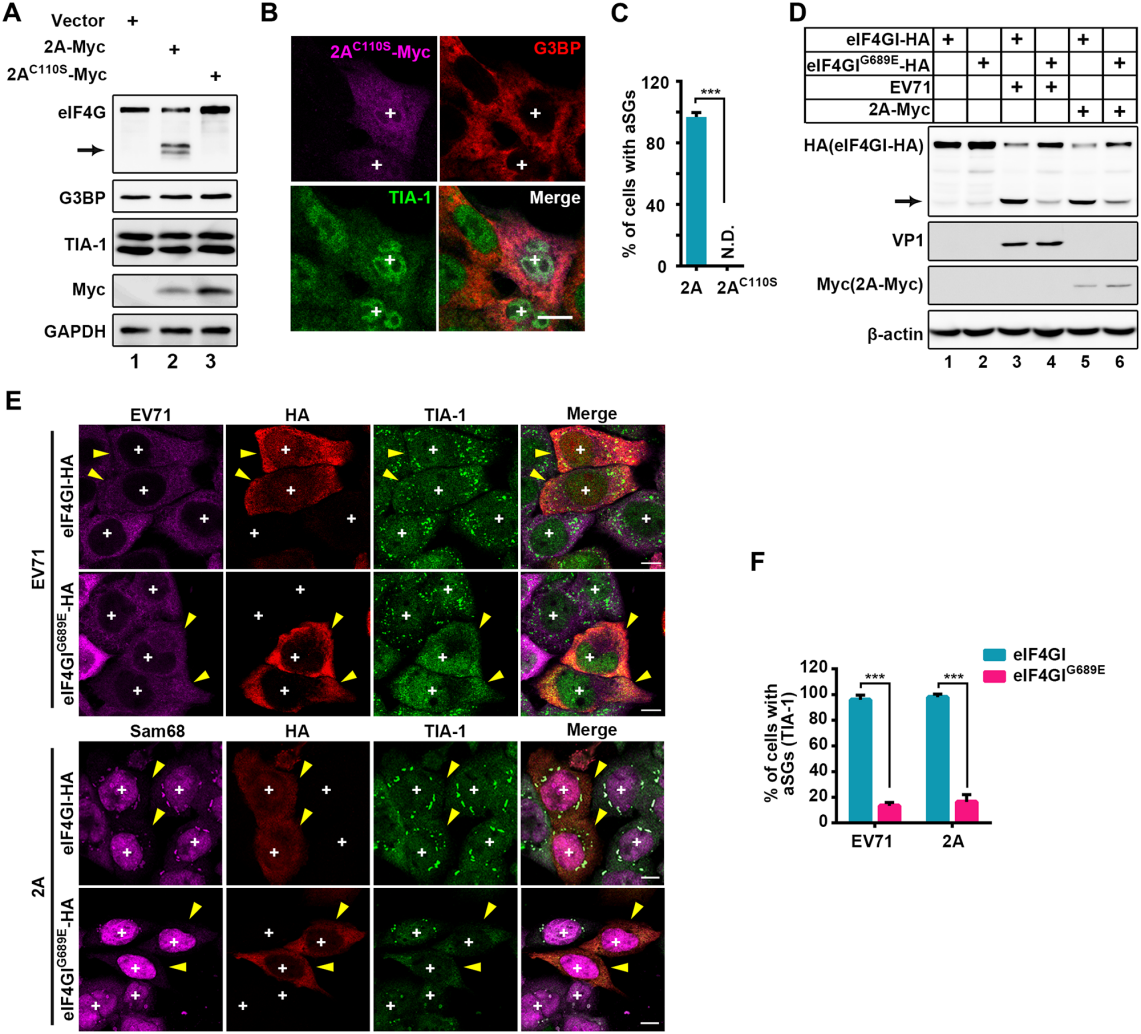
2A induces aSGs by cleaving eIF4GI. (A-C) Effects of protease activity of 2A on aSG formation. HeLa cells were transiently transfected with empty vector, Myc-tagged 2A, or 2A^C110S^ for 24 h and then subjected to WB; GAPDH was the sample loading control. Arrow indicates the N-terminal cleavage products of eIF4G (A). IF assay of aSG formation in 2A^C110S^-expressing cells (B) and quantitative analysis of the 2A- (in S2B Fig) or 2A^C110S^-expressing cells with aSGs in B. N.D., not detected. n = 3, 240 cells/condition were counted, mean±SD; ***p<0.001 (C). (D-F) Effects of cleavage of eIF4GI on aSG formation. HeLa cells were transfected with HA-tagged eIF4GI or eIF4GI cleavage-resistant mutant (eIF4GI^G689E^) for 24 h, followed by EV71 infection or 2A transfection. The cleavage of eIF4GI and eIF4GI^G689E^ was analyzed by WB assay, arrow indicates the C-terminal cleavage products of eIF4GI (D), and the formation of aSGs was viewed by IF assay (E). Quantitation analysis and comparison of eIF4GI- or eIF4GI^G689E^-expressing cells with aSGs (TIA-1 foci) among EV71-infected or 2A-expressing cells in E. n = 3, 240 cells/condition were counted, mean±SD; ***p<0.001 (F). “+” indicates the 2A/2A^C110S^-expressing or EV71-infected cells, and yellow arrows indicate the HA-tagged eIF4GI/eIF4GI^G689E^-expressing cells. Scale bars, 10 μm. See also S3 Fig.

Next, we sought to determine the role 2A plays in aSG formation. Since 2A is an important viral protease, we determined whether 2A protease activity is required for the formation of aSGs. Previous studies showed that a 2A mutant, 2A^C110S^, was catalytically inactive [30,31]. We also found that 2A^C110S^ indeed lost the ability to cleave eIF4G (Fig 2A); subsequently, 2A^C110S^ also lost the ability to induce aSG formation (Fig 2B and 2C), demonstrating that 2A protease activity is essential for aSG formation.

Furthermore, based on three facts, (1) both eIF4G and PABP were cleavage substrates of 2A and excluded from 2A-induced aSGs; (2) both eIF4GI and eIF4GII play similar functions in translation initiation, but cleavage of eIF4GI by 2A of picornaviruses is more sensitive than cleavage of eIF4GII [32,33]; (3) the mRNA level of eIF4GI was previously shown to be much higher than that of eIF4GII in mammalian cells [34], therefore, we hypothesized that cleavage of eIF4GI/PABP by 2A might be critical for 2A-induced aSG formation.

To this end, HeLa cells transiently expressing eIF4GI^G689E^ (a 2A cleavage-resistant eIF4GI mutant) [35] or PABP^M490P/Q540N^ (a 2A and 3C double-resistant mutant) [36,37] were infected with EV71 or treated with 2A. We found that eIF4GI^G689E^ and PABP^M490P/Q540N^ were indeed resistant to 2A cleavage (Fig 2D and S3A Fig), and eIF4GI^G689E^ expression dramatically blocked EV71- or 2A-induced aSG formation (Fig 2E and 2F, cells marked by “yellow arrow” and “+” indicate eIF4GI^G689E^ expression with EV71 infection or 2A expression), whereas PABP^M490P/Q540N^ expression had no effect on the formation of aSGs (S3B and S3C Fig), suggesting that cleavage of eIF4GI is critical for the formation of 2A-induced aSGs.

### 3C is dispensable for the blockage of tSG formation during EV71 infection

**Fig 3:**
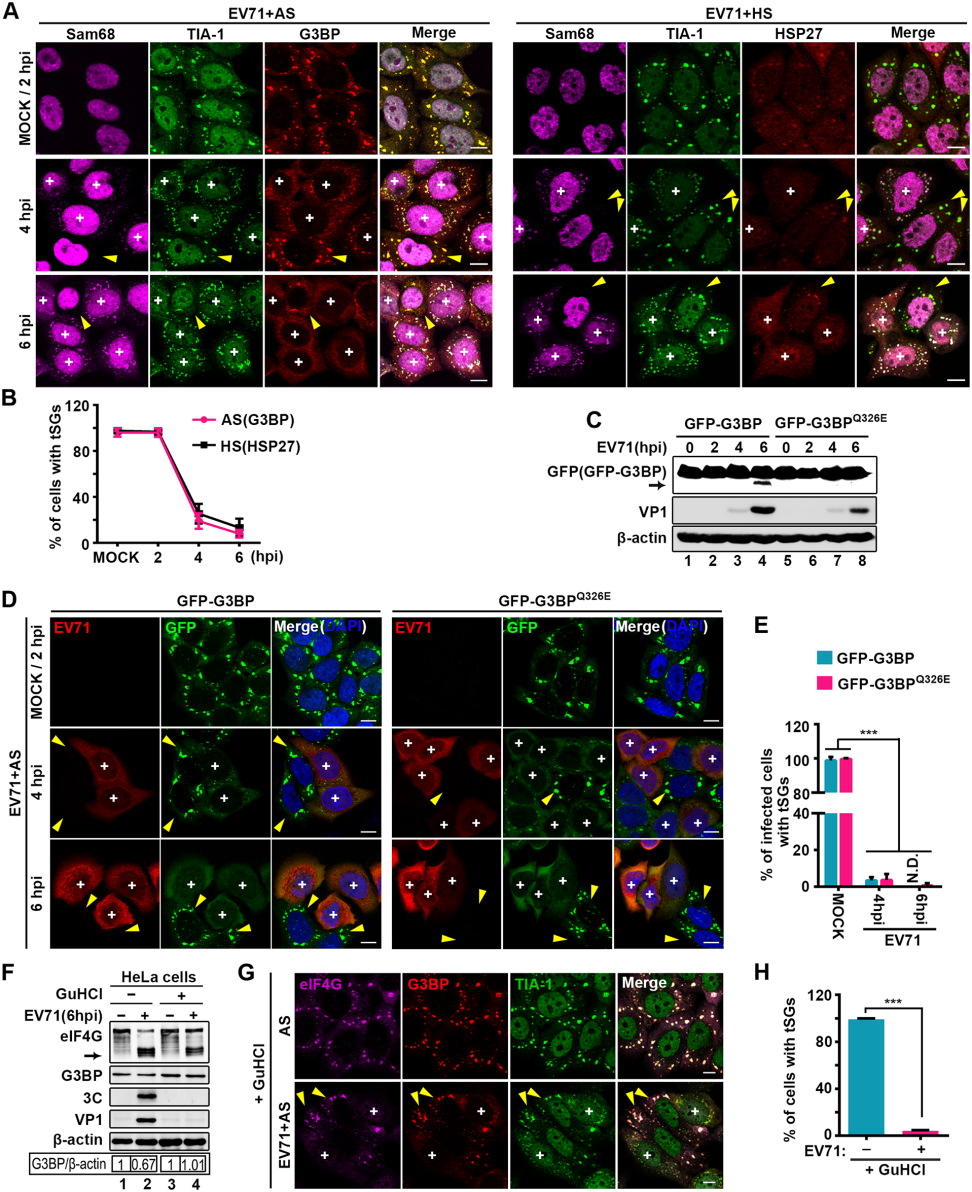
EV71 infection blocks tSG formation independent of 3C. (A) EV71 effects on AS- and HS-induced tSG formation. HeLa cells were mock-infected or infected with EV71 (MOI = 10) for consecutive times and treated with AS or HS for 1 h prior to fixation. Cells were then stained with Sam68 (magenta), TIA-1 (green), G3BP (red), or HSP27 (red) as indicated. (B) Quantitative analysis of cells with tSGs in A. tSGs were marked by G3BP or HSP27. n = 3, 300 cells/condition were counted, mean±SD. (C) GFP-G3BP- or GFP-G3BP^Q326E^-HeLa cells were infected with EV71 as in A. Cells were harvested and analyzed via WB. VP1 served as an EV71 marker, and β-actin was the loading control. Full-length GFP-G3BP and N-terminal cleavage products of GFP-G3BP were detected by antibody against GFP, and arrow indicates the N-terminal cleavage products of GFP-G3BP. (D) GFP-G3BP- or GFP-G3BP^Q326E^-HeLa cells were treated and harvested as in A. Cells were then stained with EV71 (red), GFP-G3BP and GFP-G3BP^Q326E^ (green) were markers of tSGs. (E) Quantitative analysis of EV71-infected cells with tSGs in D. n = 3, 300 cells/condition were counted, mean±SD; ***p<0.001. N.D., not detected. (F) HeLa cells were mock-infected or infected with EV71 in the presence or absence of GuHCl for 6 h as indicated and subjected to WB. Endogenous eIF4G and G3BP were detected by antibodies against eIF4G and G3BP. 3C and VP1 expression served as markers of EV71; β-actin was the sample loading control. (G) HeLa cells were mock-infected or infected with EV71 in the presence of GuHCl for 6 h and treated with AS for 1 h before harvest. Cells were then stained with eIF4G (magenta), G3BP (red), and TIA-1 (green). (H) Quantitative analysis of EV71-infected cells with tSGs (marked by G3BP) in G. n = 3, 240 cells/condition were counted, mean±SD; ***p<0.001. “+” indicates the EV71-infected cells, and yellow arrows indicate the uninfected cells. Scale bars, 10 μm. See also S4 Fig.

Because EV71 induced the formation of aSGs that were distinct from tSGs, we sought to determine whether tSG formation could be blocked during EV71 infection. We infected HeLa cells with EV71, treated them with AS or HS for 1 h prior to fixation at the indicated times, and stained them with antibodies against Sam68 (a marker of EV71-infected cells), TIA-1 (a marker of both tSGs and aSGs), G3BP (a marker of tSGs), or HSP27 (a marker of HS-induced tSGs). When cells were mock- or EV71-infected for 2 h, tSGs marked by G3BP and HSP27 were observed in all the cells upon treatment with AS or HS. As infection proceeded, tSGs were observed in only 20% of the cells at 4 hpi and 10% of the cells at 6 hpi, despite treatment with AS or HS (Fig 3A and 3B); similar results were also observed in EV71-infected RD cells (S4A and S4B Fig), suggesting that EV71 infection blocks the formation of AS- or HS-induced tSGs. However, EV71 infection had no effect on the AS- or HS-activated phosphorylation of eIF2α (S4C Fig), indicating that blockage of tSG formation by EV71 infection is not due to inhibition of eIF2α phosphorylation.

Next, we sought to determine how EV71 blocks tSG formation. Previous studies reported that 3C protease of picornaviruses inhibited tSG formation by cleavage of G3BP, and G3BP^Q326E^ (a 3C cleavage-resistant mutant of G3BP) restored SG formation competency in picornavirus-infected cells [21,38]. We confirmed that GFP-G3BP^Q326E^ was indeed cleavage-resistant upon EV71 infection (Fig 3C) or 3C expression (S4D Fig, left panel), and GFP-G3BP^Q326E^ rescued tSG formation in 3C-expressing cells (S4D Fig, right panel). To determine whether EV71 inhibits tSG formation in a manner similar to that of previous reported picornaviruses, we assessed tSG formation in the presence of EV71 infection, GFP-G3BP or GFP-G3BP^Q326E^ expression plus AS. At 2 hpi with EV71, GFP-G3BP and GFP-G3BP^Q326E^ localized to AS-induced tSGs. As infection proceeded, AS-induced tSG formation was blocked in EV71-infected cells despite expression of GFP-G3BP^Q326E^ (Fig 3D and 3E). Similar results were observed in EV71-infected RD cells expressing GFP-G3BP or GFP-G3BP^Q326E^ (S4E Fig), suggesting that 3C cleavage of G3BP is dispensable for the inhibition of tSG formation during EV71 infection.

To confirm whether 3C protease is required for the inhibition of tSG formation during EV71 infection, we used guanidine hydrochloride (GuHCl, an ATPase inhibitor) to suppress viral replication to an extremely low level in HeLa and RD cells [39,40]. Under these conditions, viral proteins were undetectable, and the level of full-length G3BP in EV71-infected cells was comparable with that in uninfected cells, but eIF4G was remarkably cleaved (Fig 3F and S4F Fig), as reported previously [21,41], indicating that 3C cannot work and the cleavage of eIF4G can be used to distinguish the infected cells. However, the AS-induced tSG formation was still inhibited in EV71-infected cells (Fig 3G and 3H and S4F Fig). Taken together, our results demonstrate that the blockage of tSG formation during EV71 infection is not due to 3C protease.

### 2A blocks tSG formation

**Fig 4:**
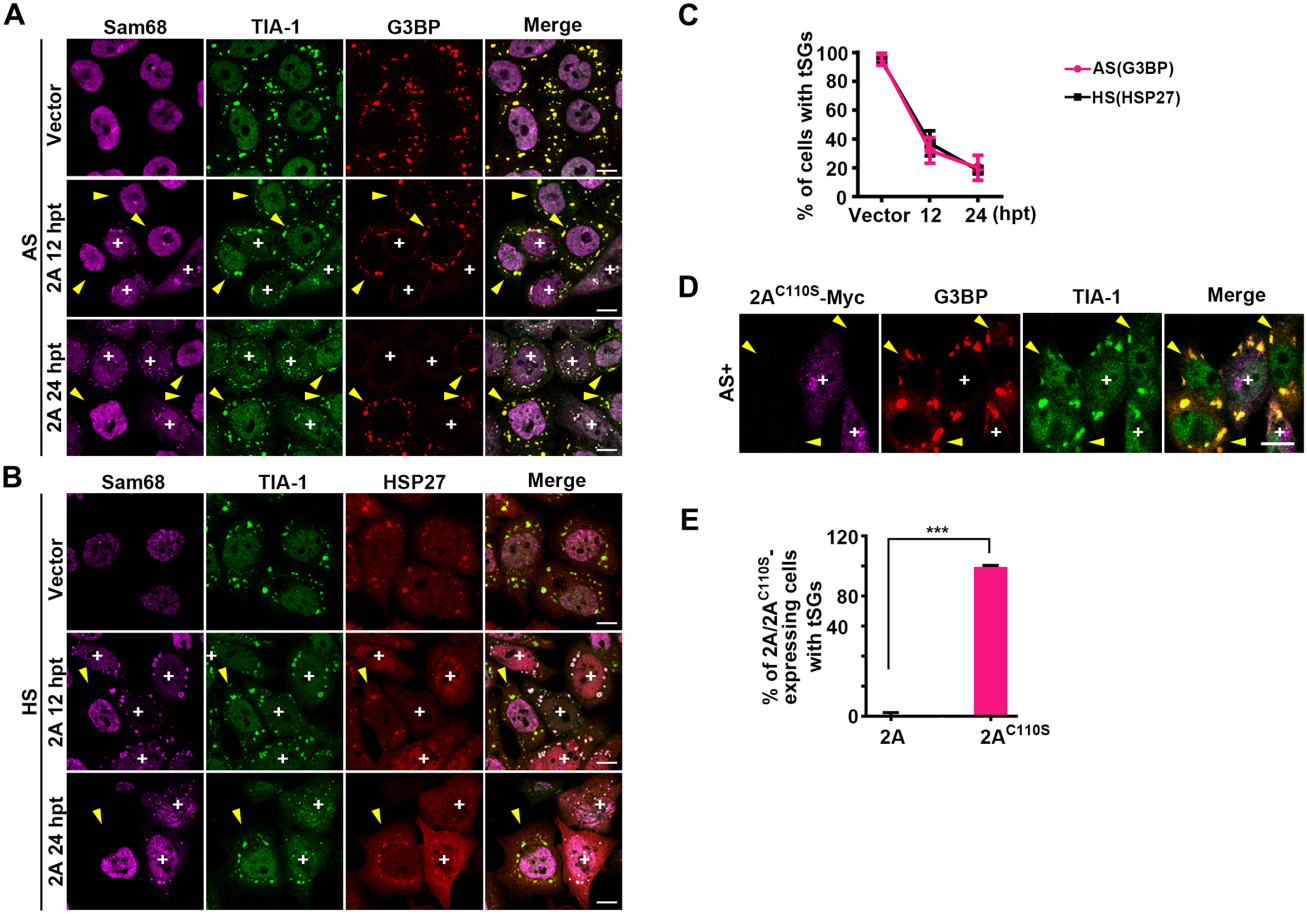
2A protease can block tSG formation. (A and B) HeLa cells were transfected with 2A for 12 h and 24 h or transfected with empty vector for 24 h as a control, followed by 1 h treatment with AS or HS, and stained with Sam68 (magenta), TIA-1 (green), G3BP (red), or HSP27 (red) as indicated. (C) Quantitative analysis of cells with tSGs in A and B as marked by G3BP or HSP27. n = 3, 240 cells/condition were counted, mean±SD. (D-E) Effects of protease activity of 2A on tSG blockage. HeLa cells were transfected with Myc-tagged 2A^C110S^ for 24 h and treated with AS for 1 h. tSG formation was viewed by IF assay (D). Quantitation analysis of 2A-expressing cells (in A, 24 hpt) or 2A^C110S^-expressing cells (in D) with SGs. n = 3, 240 cells/condition were counted, mean±SD; ***p<0.001 (E). “+” indicates the 2A/2A^C110S^-expressing cells, and yellow arrows indicate the cells without 2A/2A^C110S^ expression. Scale bars, 10 μm. See also S5 Fig.

Since 3C was dispensable for the blockage of tSG formation, and 2A-induced aSGs did not contain tSG components such as G3BP and eIF4G, we hypothesized that 2A plays a critical role in inhibiting tSG formation. To validate this possibility, we examined tSG formation induced by AS or HS in the presence of 2A (Sam68 foci indicate 2A-expressing cells) and found that AS- or HS-induced tSGs (marked by G3BP or HSP27) appeared in 96% of empty vector-transfected cells but appeared in only 35% of 2A-transfected cells at 12 h and in less than 20% of 2A-transfected cells at 24 h (Fig 4A–4C). 2A-expressing cells all failed to form tSGs (Fig 4A and 4B, “+” indicates expression of 2A). Similar results were observed in 2A-transfected RD cells (S5A and S5B Fig). Furthermore, 2A expression had no effect on AS- or HS-activated eIF2α phosphorylation (S5C Fig), suggesting that 2A blocks tSG formation without inhibiting eIF2α phosphorylation, which is consistent with those in EV71 infection conditions (S4C Fig).

To confirm that 2A indeed plays a critical role in the inhibition of tSG formation during EV71 infection, we expressed Myc-tagged 2A in HeLa cells stably expressing GFP-G3BP^Q326E^ and then treated cells with AS and found that 2A, but not 3C, blocked the formation of tSGs (S5D and S5E Fig), suggesting that 2A, but not 3C, plays a critical role in the inhibition of tSG formation during EV71 infection. In addition, we found that 2A^C110S^ also lost the ability to block the formation of AS-induced tSGs (Fig 4D and 4E), suggesting that 2A protease activity is essential for its blockage of tSG formation. Having found that eIF4GI^G689E^ blocked aSG formation, we further analyzed whether eIF4GI^G689E^ could restore tSG formation in the presence of AS. To our surprise, we found that 2A still blocked tSG formation in spite of expression of eIF4GI^G689E^ (S5F and S5G Fig), suggesting that the blockage of tSG formation is not due to cleavage of eIF4GI by 2A.

### Protease activity of 2A is essential for the blockage of tSG formation and induction of aSG formation during EV71 infection

To further elucidate the critical role of 2A protease activity in blocking tSG formation and inducing aSG formation during EV71 infection, we generated recombinant EV71 with the 2A^C110S^ mutation (EV71-2A^C110S^). Since 2A is important for the viral life cycle, the replication activity of EV71-2A^C110S^ is much lower (50-fold less in viral titer) than that of EV71 and VP1 expression of EV71-2A^C110S^ was also much lower than that of EV71 (S6A Fig). Correspondingly, EV71-2A^C110S^ could not shut off cellular translation as quickly as EV71 (S6B Fig).

**Fig 5:**
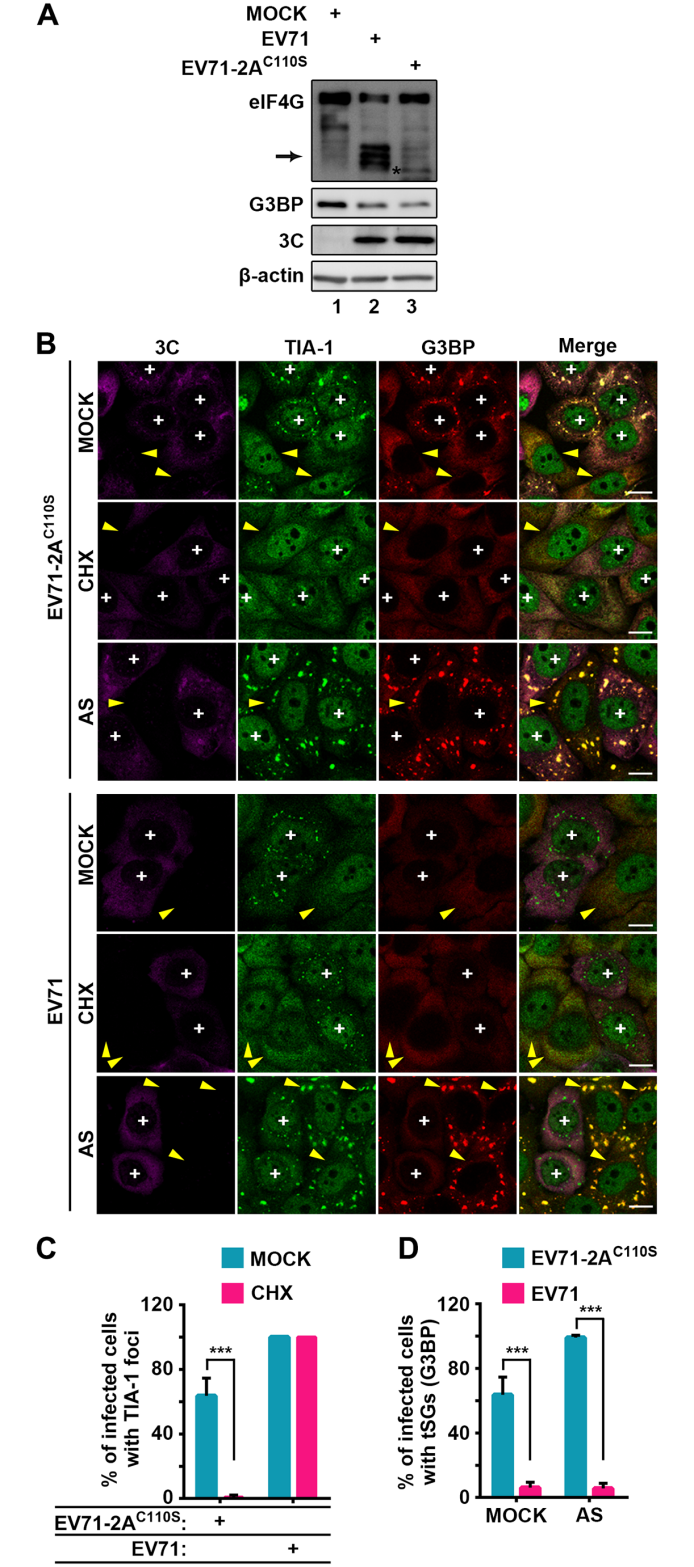
Protease activity of 2A is required for tSG blockage and aSG induction during EV71 infection. (A) Confirmation of 2A protease activity ablation in EV71-2A^C110S^. HeLa cells were mock-infected or infected with EV71 (MOI = 0.5) or recombinant virus encoding 2A^C110S^ (EV71-2A^C110S^, MOI = 3) and subjected to WB for detection of eIF4G and G3BP cleavage. 3C indicated EV71 replication, and β-actin was the sample loading control. Arrow indicates eIF4G products cleaved by 2A, and “*” indicates eIF4G products cleaved by caspases [32,61]. (B) HeLa cells were infected with EV71 or EV71-2A^C110S^ as in A, and then mock-treated or treated with CHX or AS for 1 h before fixation. Cells were then stained with 3C (magenta), TIA-1 (green), HSP27 (red), or G3BP (red) as indicated. “+” indicates the infected cells, and yellow arrows indicate the uninfected cells. (C) Quantitative analysis of infected cells with TIA-1 foci in B (mock panel and CHX panel). n = 3, 300 cells/condition were counted, mean±SD; ***p<0.001. (D) Quantitative analysis of infected cells with tSGs (G3BP) in B (mock panel and AS panel). n = 3, 300 cells/condition were counted, mean±SD; ***p<0.001.Scale bars, 10 μm. See also S6 Fig.

Therefore, we infected cells with a higher MOI of EV71-2A^C110S^ and a lower MOI of EV71 and found that when the 3C protein levels and G3BP cleavage levels were comparable, eIF4G was no longer cleaved in EV71-2A^C110S^-infected cells (Fig 5A). Correspondingly, EV71-2A^C110S^ infection failed to induce aSG formation; instead, EV71-2A^C110S^ induced the formation of tSGs containing G3BP and TIA-1 in about 65% of infected cells, which could be completely dispersed by CHX (Fig 5B and 5C). With additional AS or HS treatment, all the EV71-2A^C110S^-infected cells formed tSGs (Fig 5B and 5D and S6C and S6D Fig). Taken together, these data demonstrate that EV71 infection induces tSG formation independent of 2A protease activity, but 2A inhibits EV71-induced tSG formation and induces aSG formation.

### EV71 induces tSG formation via the PKR-eIF2a pathway

**Fig 6:**
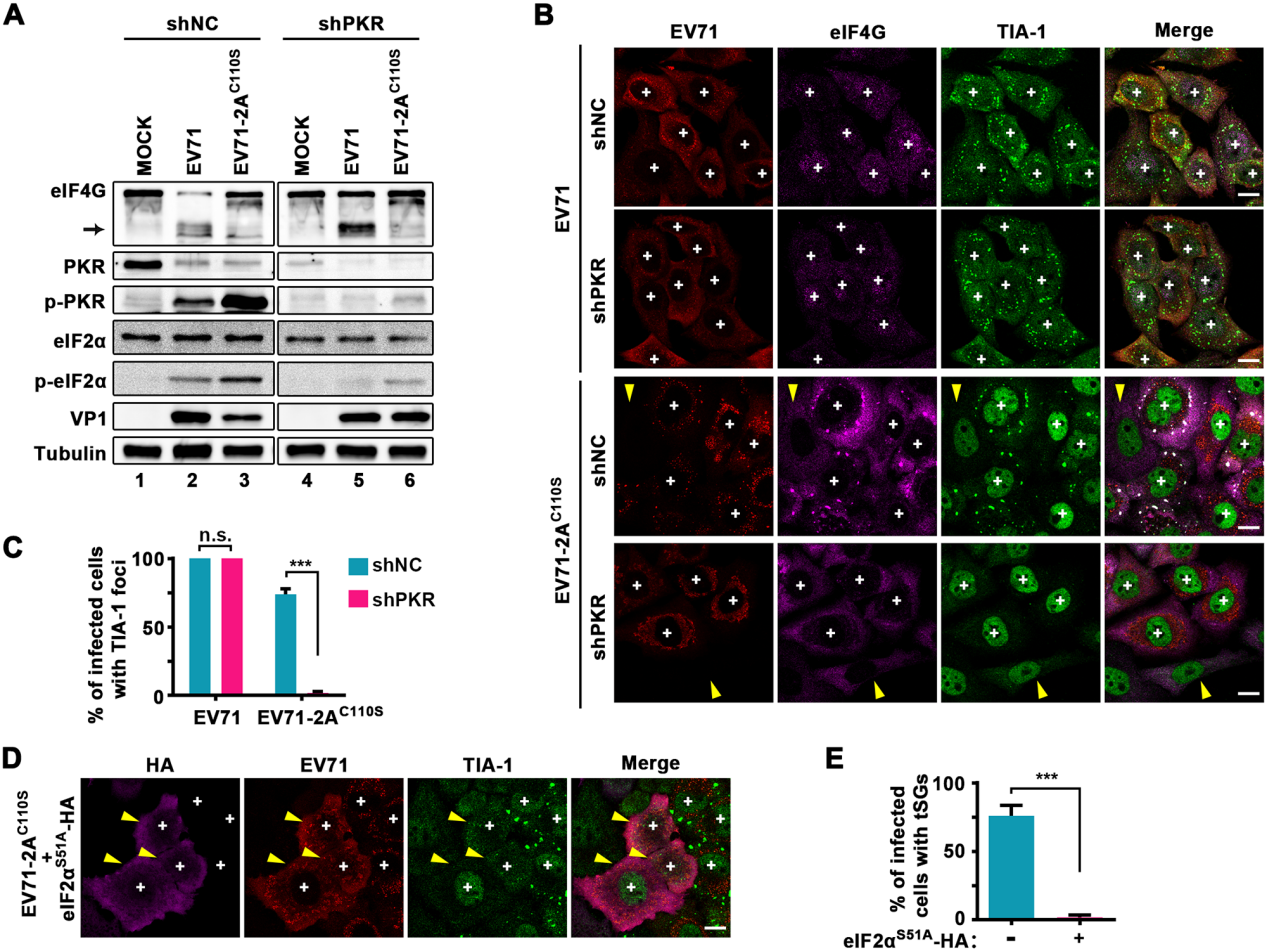
EV71 induces tSG formation via the PKR-eIF2α pathway. (A) KD of PKR effects on EV71-2A^C110S^-induced phosphorylation of PKR and eIF2α. shNC- and shPKR-HeLa cells were infected with EV71 (MOI = 1, as control) or EV71-2A^C110S^ (MOI = 3) for 12 h and subjected to WB. Tubulin served as a loading control. (B) KD of PKR effects on EV71-2A^C110S^-induced tSG formation. Cells were infected as in A and subjected to IF assay. “+” indicates the infected cells, and yellow arrows indicate the uninfected cells.(C) Quantitative analysis of cells with TIA-1 foci in B. n = 3, 300 cells/condition were counted, mean±SD; n.s., no statistical significance, ***p<0.001. (D) Phosphorylation of eIF2α effects on EV71-2A^C110S^-induced tSG formation. HeLa cells were transfected with HA-tagged eIF2α^S51A^ for 24 h, and then infected with EV71-2A^C110S^ (MOI = 3) for 12 h. “+” indicates the infected cells, and yellow arrows indicate the eIF2α^S51A^-HA-expressing cells. (E) Quantitative analysis of the cells (in D) with tSGs among HA-positive or HA-negative cells. n = 3, 240 cells/condition were counted, mean±SD; ***p<0.001. Scale bars, 10 μm.

We next sought to determine how EV71 infection induces tSG formation. Since EV71 is a positive-sense single-stranded RNA virus, it generates significant amounts of viral replication intermediate dsRNAs during replication. And the dsRNAs commonly activate PKR to phosphorylate eIF2α, which results in tSG assembly [42]. To determine whether EV71-2A^C110S^ induces tSG formation via the PKR-eIF2α pathway, we generated HeLa cells with stable knockdown (KD) of PKR (shPKR-HeLa cells) and infected them with EV71 or EV71-2A^C110S^. We found that KD of PKR decreased the phosphorylation levels of PKR and eIF2α in EV71- or EV71-2A^C110S^-infected cells (Fig 6A). In shPKR-HeLa cells, the formation of EV71-induced aSGs was not affected, but the formation of EV71-2A^C110S^-induced tSGs was blocked (Fig 6B and 6C). Furthermore, expression of eIF2α^S51A^ blocked the formation of EV71-2A^C110S^-induced tSGs but not EV71-induced aSGs (Fig 6D and 6E). Taken together, these results suggest that, unlike aSGs, tSGs are induced via the PKR-eIF2α pathway by viral dsRNAs during EV71 infection.

### 2A transforms from tSGs to aSGs to benefit viral translation

**Fig 7:**
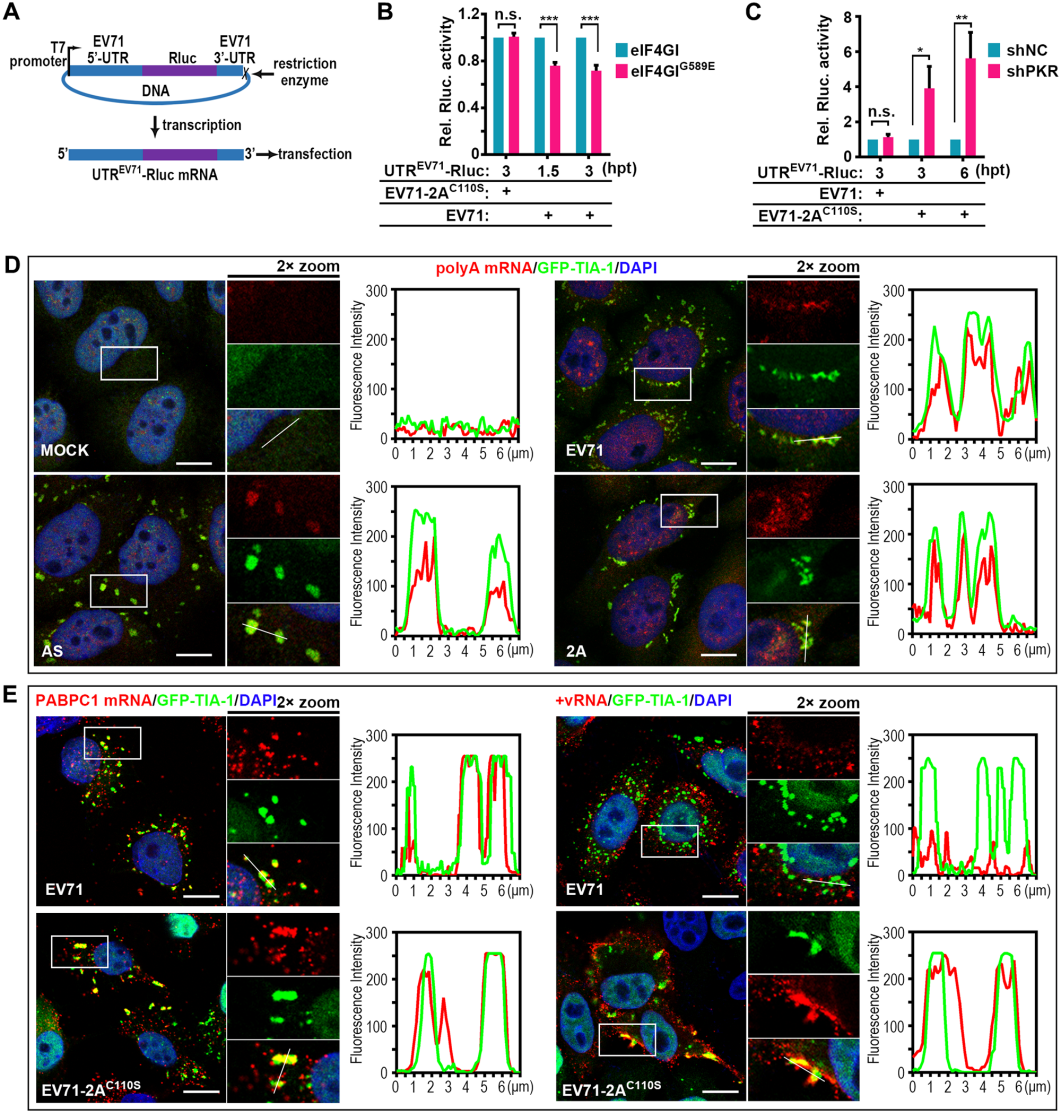
2A induces aSGs to stall host mRNAs and release viral mRNAs. (A-C) Effects of EV71-induced aSGs and EV71^C110S^-induced tSGs on viral translation. Graphic description of EV71-UTR driven Renilla luciferase reporter expression (A) and reporter assays in HeLa cells (B and C). Cells were infected with EV71 for 3h to induce aSG formation or infected with EV71-2A^C110S^ for 6h to induce tSG formation before transfection with UTR^EV71^-Rluc mRNAs, and the luciferase activity was measured at indicated time post-transfection. The EV71-2A^C110S^-infected eIF4GI/eIF4GI^G689E^-HA-HeLa cells and EV71-infected shNC/shPKR-HeLa cells were negative controls. n = 3, mean±SD, n.s., no statistical significance; *p<0.05; **p<0.01; ***p<0.001. (D) FISH assays of total polyA mRNA colocalized to TIA-1 foci in GFP-TIA-1-HeLa cells after mock treatment or treatment with AS, EV71, or 2A. GFP-TIA-1 (green), polyA mRNAs (red), and nuclei (blue) are shown. The fluorescence intensity profile of GFP-TIA-1 (green) and polyA mRNA (red) was measured along the line drawn on a 2× zoom panel by Leica Application Suite Advanced Fluorescence Lite. (E) FISH assays of GFP-TIA-1-HeLa cells infected with EV71 or EV71-2A^C110S^. PABPC1 mRNA (left panel) and EV71 mRNA (+vRNA; right panel) were monitored. The fluorescence intensity profile of GFP-TIA-1 (green) and +vRNA/PABPC1 mRNA (red) was measured along the line drawn on a 2× zoom panel by Leica Application Suite Advanced Fluorescence Lite. Scale bars, 10 μm. See also S7 Fig.

Next, we sought to determine why 2A blocks tSG formation but induces aSG formation. We hypothesized that it is a strategy by which the virus facilitates its own translation. Thus, we generated a Renilla luciferase mRNA reporter, the translation of which is driven by EV71-UTR to mimic EV71 translation (UTR^EV71^-Rluc) (Fig 7A). We found that eIF4GI^G689E^ did not influence translation of UTR^EV71^-Rluc in EV71-2A^C110S^-infected cells, but once aSG formation was inhibited by eIF4GI^G689E^ in EV71-infected cells, the translation efficacy of UTR^EV71^-Rluc was dramatically decreased (Fig 7B), suggesting that aSG formation benefits EV71 translation. Conversely, the translation efficacy of UTR^EV71^-Rluc was not influenced by KD of PKR in EV71-infected cells, but increased at least three-fold when tSG formation was inhibited by KD of PKR in EV71-2A^C110S^-infected cells (Fig 7C), suggesting that tSGs inhibit EV71 translation.

Next, we sought to determine how aSGs benefit viral translation. We hypothesized that different mRNAs are sequestered in aSGs and tSGs to regulate viral translation. First, using poly(A) fluorescence in situ hybridization (FISH) assays, we confirmed that numerous mRNAs were present in the EV71- or 2A-induced aSGs and in the AS-induced tSGs (Fig 7D). Second, using RNA FISH assays, we evaluated the localization of viral mRNAs (positive-strand RNA, +vRNA) and cellular PABPC1 mRNAs (with high TIA-1 affinity) [43] in HeLa cells stably expressing GFP-TIA-1. In EV71-infected cells, PABPC1 mRNAs, but not +vRNA, efficiently localized in the aSGs (Fig 7E, top panel). In EV71-2A^C110S^-infected cells, both PABPC1 mRNAs and +vRNA efficiently localized in the tSGs (Fig 7E, bottom panel). Furthermore, we generated a Renilla luciferase mRNA reporter, the translation of which is driven by PABPC1-UTR to mimic PABPC1 translation (UTR^PABPC1^-Rluc) (S7A Fig). We found whether the aSGs was blocked by eIF4GI^G689E^ in EV71-infected cells or the tSGs was blocked by KD of PKR in EV71-2A^C110S^-infected cells, the translation efficacy of UTR^PABPC1^-Rluc mRNA increased (S7B and S7C Fig), suggesting that both aSGs and tSGs inhibit PABPC1 mRNA translation. Taken together, our results suggest that tSGs stall both cellular and viral mRNAs to shut down overall translation, resulting in the inhibition of viral translation; however, 2A of EV71 blocked tSG formation but induced aSG formation to facilitate viral translation by stalling only cellular mRNAs.

### 2A-mediated induction of aSG formation and blockage of tSG formation are common among *Picornaviridae*

**Fig 8:**
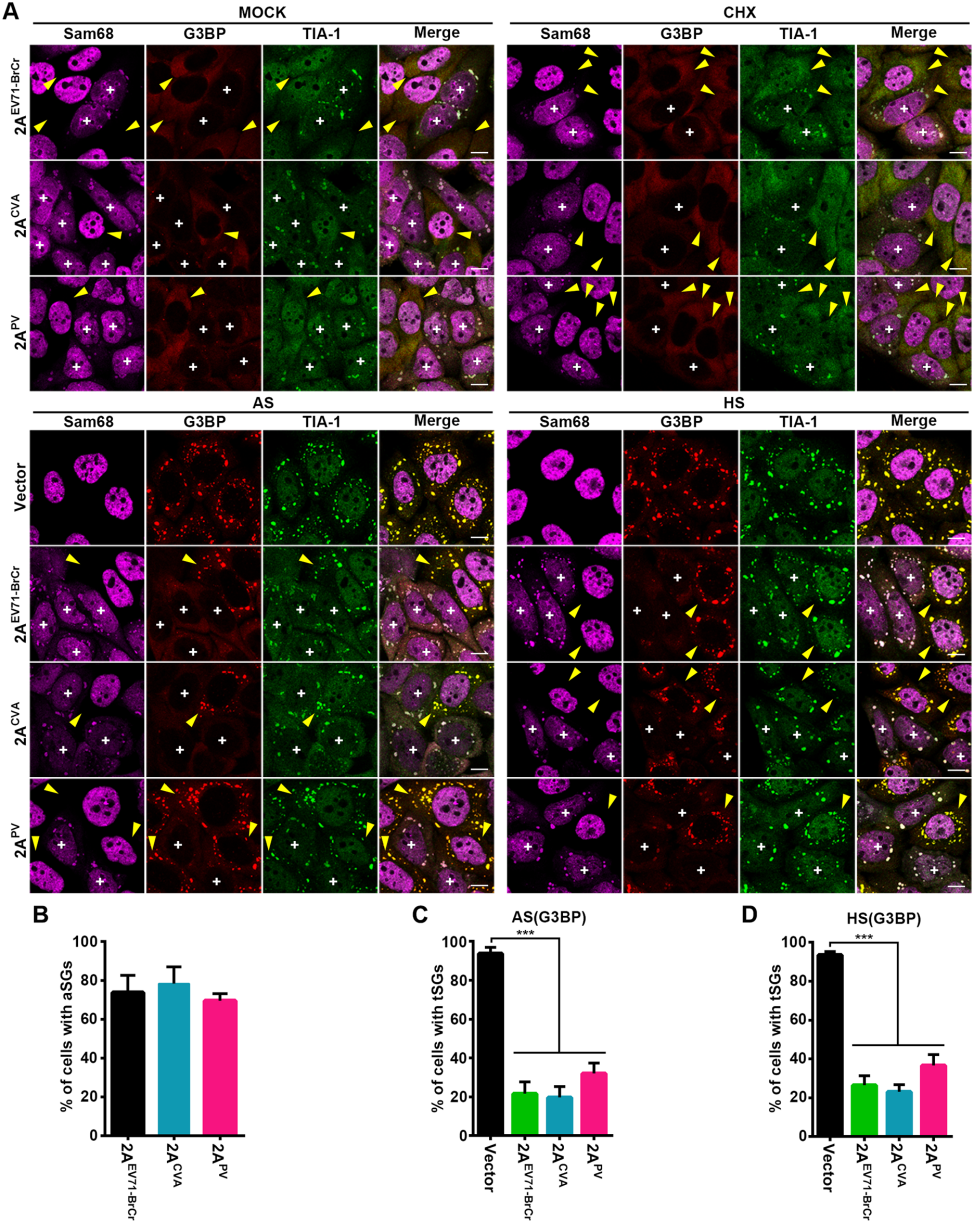
2A-induced aSG formation and 2A-inhibited tSG formation are common among picornaviruses. (A) HeLa cells were transiently transfected with 2A of EV71-BrCr, CVA, or PV for 24 h and then mock-treated or treated with CHX (top panel), AS, or HS (bottom panel). Cells were stained with Sam68 (indicator of 2A expression and aSGs), G3BP (marker of tSGs), and TIA-1. “+” indicates the 2A-expressing cells, and yellow arrows indicate cells without 2A expression. (B) Quantitative analysis of cells with aSGs (marked by TIA-1) upon mock treatment (top and left panel in A). n = 3, 240 cells/condition were counted, mean±SD. (C-D) Quantitative analysis of cells with tSGs marked by G3BP upon AS (C) and HS treatment (D). n = 3, 240 cells/condition were counted, mean±SD; ***p<0.001. Scale bars, 10 μm. See also S8 Fig.

The function of the 2A protease is highly conserved among *Picornaviridae*. Thus, we sought to determine whether its role in SG formation regulation is conserved among picornaviruses. We used Sam68 cytoplasmic re-localization as a marker of 2A expression in IF assays. Indeed, expression of 2A of EV71-BrCr, PV, and coxsackievirus A (CVA) also triggered Sam68 and TIA-1 to form aSGs that were devoid of G3BP and resistant to CHX (Fig 8A, upper panel, and Fig 8B). Furthermore, 2A of these picornaviruses also blocked AS- and HS-induced tSG formation (Fig 8A, lower panel, and Fig 8C and 8D). Similarly, 2A of these picornaviruses cleaved eIF4GI but not eIF4GI^G689E^ (S8A Fig), and 2A of these picornaviruses also failed to induce the formation of aSGs in eIF4GI^G689E^-expressing cells (S8B and S8C Fig). Taken together, these findings suggest that although different picornaviruses may use different mechanisms to alter host cell function, the effect of 2A on the regulation of SGs is common.

## Discussion

In previous studies, TIA-1 foci have been observed during picornavirus infection; however, the essence of these aggregates was ambiguous and controversial. The 2A-induced TIA-1 foci were thought to be tSGs [22]. We also found that 2A-induced TIA-1 foci share some features with tSGs, such as they are dynamic and adjacent to p-bodies (Fig 1C and S2C Fig), but they are different from tSGs and thus named them aSGs. The conclusion of aSGs different from tSGs is supported by multiple lines of evidence. First, the aSGs contained TIA-1, TIAR, and Sam68 but were devoid of G3BP, a series of eIFs and viral mRNA (S1A–S1G and S2B Figs and Fig 7E). Second, the formation of aSGs was independent of PKR activation (Fig 6B and 6C) and the phosphorylation of eIF2α (Fig 1D and 1E). Third, the aSGs could not be disassembled by CHX (Fig 1F and 1G). Fourth, EV71-2A^C110S^-induced TIA-1 foci were tSGs, which contained TIA-1, G3BP, eIF4G, and viral mRNA, and could be completely dispersed by CHX, and the formation of which was dependent on PKR activation and the phosphorylation of eIF2α (Figs 5B, 6B and 7E).

Although both eIF4G and PABP were cleavage substrates of 2A, the cleavage efficiency of PABP is much lower than that of eIF4G [44]. We found that the only eIF4GI^G689E^ blocked the aSGs formation, indicating that 2A induces aSG formation by cleaving eIF4GI (Fig 2E and 2F and S3A–S3C Fig). PABP is also a cleavage substrate of 3C, but expression of 3C is unable to induce aSG formation (S2A Fig). Therefore, we thought that even if the cleavage of PABP is more robust, cleavage of PABP should not result in aSG formation. We also found that 2A-induced aSGs are a common phenomenon in picornaviruses (Fig 8), and the dynamics of TIA-1 in aSGs were similar to those of TIA-1 in tSGs (Fig 1C). Thus, it is possible that aSGs are also non-membrane-bound cellular compartments and formed via liquid-liquid phase separation (LLPS), as is the case for tSGs. Although the details of LLPS are not clear, previous studies have shown that the high concentration of RBPs, which contain intrinsically disordered regions (IDRs), triggers LLPS [45–47]. When the concentration of IDR-containing RBPs reaches a certain level, the LLPS of these RBPs can be spontaneously initiated by IDR-mediated interaction, thus promoting tSG formation. Furthermore, during molecular crowding, IDR-containing RBPs can initiate LLPS at lower protein levels [46]. The expression of 2A could shuttle many nuclear IDR-containing RBPs, including hnRNPs, TDP-43, HuR, and Sam68, to the cytoplasm [14,48–51], thus resulting in increased concentrations of IDR-containing RBPs in the cytoplasm. Furthermore, the cleavage of eIF4GI by 2A leads to the accumulation of stalled PICs, thus resulting in molecular crowding. In cells expressing eIF4GI^G689E^ and 2A, translation is initiated, molecular crowding is inhibited [35,52], and tSG formation is blocked (S5F and S5G Fig), and aSGs are therefore unable to form (Fig 2E and 2F and S8 Fig).

In principle, the PICs rapidly exchange between tSGs and polyribosomes, the addition of CHX during AS and HS treatment inhibits the dissociation of polyribosomes and results in the disassembly of tSGs [4,5]. However, we found that aSGs could not be dissolved by CHX (Fig 1F and 1G). Furthermore, we also found that the aSGs did not contain eIF1a, eIF3a, eIF4A, eIF4E, eIF4G, and RPS3 (S1A–S1H and S2B Figs), indicating that the mRNAs in aSGs are not equipped with eIFs and 40S ribosomal subunits, and cannot participate in translation. Therefore, the mRNAs within aSGs cannot flow to polyribosomes and aSGs cannot be dissolved by CHX.

Piotrowska et al. found that poliovirus did not block HS-induced tSG formation at 4 hpi [14], but we found that both AS- and HS-induced tSG formation were inhibited at 4 hpi and 6 hpi during EV71 infection (Fig 3A and 3B and S4A and S4B Fig). We speculate that poliovirus could not block HS-induced tSGs completely at 4hpi, but as infection proceeded, poliovirus blocked HS-induced tSGs eventually. To our knowledge, we are the first to find that tSGs are totally abrogated specifically by 2A, but not by 3C. Our conclusion is supported by three lines of evidence. First, EV71 and 2A still blocked tSG formation in cells expressing 3C cleavage-resistant G3BP (G3BP^Q326E^) (Fig 3D and 3E, S4D, S4E, S5D and S5E Figs), which contradicts previous findings [21,38,49]. We speculate that most of the viruses failed to replicate in cells expressing G3BP^Q326E^, because G3BP exhibits antiviral activity against several picornaviruses [53], thus resulting in an eight-fold reduction in viral titer and tSG formation rescue [21]. Although tSG formation was inhibited in 3C-expressing cells (S4D Fig), we think this is just a phenomenon accompanying the cleavage of G3BP by 3C to inhibit the antiviral activity of G3BP in the late phase of EV71 infection, because tSG inhibition occurred much earlier than G3BP cleavage (Fig 3C and 3D). Second, the addition of GuHCl in EV71-infected cells suppressed viral replication to an extremely low level, and the protease activity of 3C could not be detected (Fig 3F and S4F Fig), but tSG formation was still blocked in infected cells (Fig 3G and 3H and S4F Fig), suggesting that 3C is not required for blocking tSG formation. Third, by using the EV71-2A^C110S^ mutant virus, in which 2A protease activity was abolished but 3C protease activity was intact (Fig 5A), we found that EV71-2A^C110S^ induced tSG formation instead of inhibiting it (Fig 5B–5D), demonstrating that 2A is indispensable for the EV71-mediated inhibition of tSG formation. We also tried to rescue recombinant EV71 with a catalytically inactive 3C mutation but failed, which may be attributed to the presence of many 3C cleavage sites in the viral polyproteins.

2A of EV71 was previously suggested to induce tSG formation, but the exact mechanism is unknown [21,22]. To our knowledge, we are the first to demonstrate that EV71 induces tSG formation through the PKR-eIF2α pathway via dsRNA, but not 2A (Fig 6); on the contrary, 2A is indispensable for the blockage of tSG formation (Figs 4 and 5 and S5 Fig). Although both induction of aSG formation and blockage of tSG formation require 2A protease activity, the molecular mechanism of 2A contribution to induction of aSG formation and blockage of tSG formation is different. The aSG formation is accompanied by the tSG blockage in 2A-expessing or EV71-infected cells (Figs 3A, 4A and 4B, S4A and S5A Figs), but inhibition of 2A-induced aSGs by expression of eIF4GI^G689E^ is unable to recover tSGs in the presence of AS (Fig 2E and 2F and S5F and S5G Fig); Furthermore, KD of PKR or expression of eIF2α^S51A^ inhibits EV71-2A^C110S^-induced tSG formation (Fig 6), but has no effect on EV71-induced aSG formation (Figs 1D, 1E, 6B and 6C), suggesting that blockage of tSG formation and induction of aSGs by virus are independent to each other, not due to turning tSGs into aSGs. The molecular mechanism of how 2A prevents tSG formation should be more complicated.

EV71-2A^C110S^-induced tSGs contained eIFs and both viral and cellular mRNAs (Figs 6B, 7D and 7E) to inhibit viral translation (Fig 7C). But EV71-induced aSGs did not contain a series of eIFs or viral mRNA (S1A–S1H Fig, Figs 6B, 7D and 7E) and instead contained selectively confined host mRNAs (Fig 7E) to facilitate viral translation (Fig 7B). The mechanism of how viral RNA avoids being recruited to the aSGs is unclear, but we thought differences of RNA-binding proteins within aSGs and tSGs result in elimination of viral RNA from aSGs. It has been reported that many cellular mRNAs also contained IRES [54], and if the binding proteins of IRES-containing cellular mRNAs are eliminated from aSGs, the aSGs should not stall these IRES-containing cellular mRNAs either.

**Fig 9:**
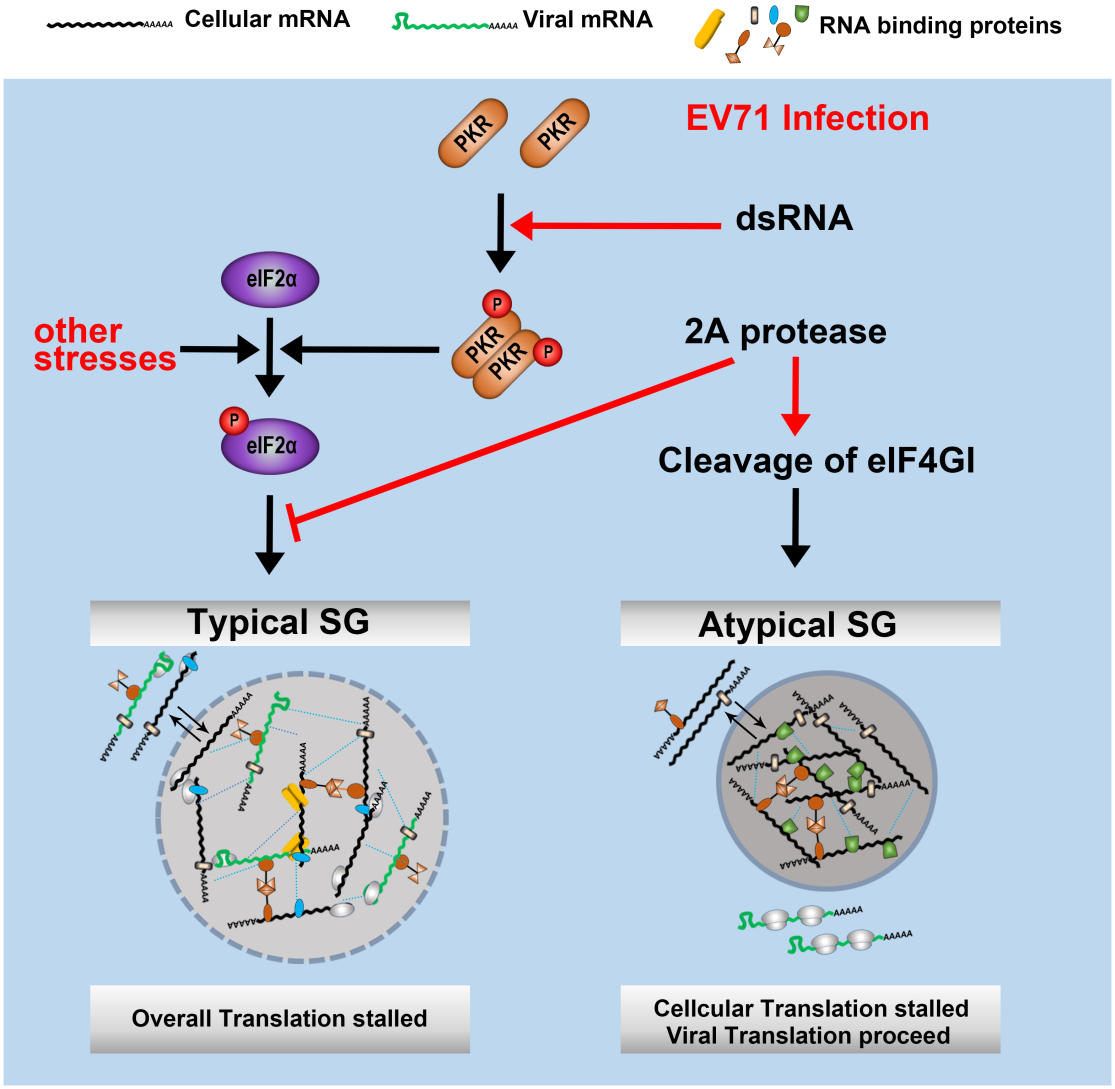
Model of SGs regulation by EV71.

In conclusion, our findings reveal the common molecular mechanisms and functions of picornavirus-mediated regulation of SG formation (Fig 9). On one hand, host cells recognize viral dsRNA via PKR during EV71 infection, which activates the PKR-eIF2α signaling cascade and results in tSG formation. Both cellular and viral mRNAs are sequestered in tSGs, which leads to an overall shutdown of translation to inhibit viral translation. On the other hand, 2A protease of EV71 blocks tSG formation drastically to remove the antiviral effects of tSG formation from host cells. Furthermore, 2A induces aSG formation by cleaving eIF4GI to sequester cellular mRNA but releases eIFs and viral mRNA, which benefit viral translation. Thus, the blockage of tSG formation and induction of aSG formation are strategies used by EV71 to survive in host cells.

Further studies are needed to reveal the complete RNA and protein composition of aSGs and the mechanisms of EV71-induced blockage of tSG formation. Likewise, the full effect of the regulation of SG formation on aspects of cell physiology other than translation control of host mRNAs should be further explored. Previous studies have suggested that persistent SG formation is linked to some neurodegenerative diseases [55–57]. Research in these areas may offer fascinating new insights into cellular function and may also yield novel therapeutic strategies in picornavirus-induced disease.

## Materials and methods

### Cell culture, infection, and transfection

Human RD (Rhabdomyosarcoma cells and were obtained from China Center for Type Culture Collection), HEK293T (Human embryonic kidney 293 cells and were obtained from China Center for Type Culture Collection), HeLa cells (Human cervical cancer epithelial cells and were obtained from China Center for Type Culture Collection), and stably expressing cells (GFP-G3BP-HeLa/RD, GFP-G3BP^Q326E^-HeLa/RD, GFP-TIA-1-HeLa) derived from HeLa or RD cells were cultured in Dulbecco’s modified Eagle’s medium (DMEM) (Gibco) supplemented with 10% fetal bovine serum (FBS) (Gibco) and 100 U/ml penicillin/streptomycin (Gibco) at 37°C and 5% CO_2_. Other stably expressing cells (eIF4GI-HA-HeLa, eIF4GI^G689E^-HA-HeLa), cells with KD of PKR (shPKR-HeLa) or negative control (shNC-HeLa) cells derived from HeLa cells were maintained in DMEM with 10% FBS, 100 U/ml penicillin/streptomycin, and 1 μg/ml puromycin (Sigma-Aldrich) at 37°C and 5% CO_2_.

For infection, HeLa or RD cells were infected with DMEM containing viruses with a multiplicity of infection (MOI) of 10 plaque-forming units (PFUs) or as indicated in the figure legends. After 1 h incubation, the medium was replaced with fresh DMEM with 4% FBS, and this time point was considered 0 hpi; cells were harvested for further analysis at 6 hpi or as indicated. For transfection, plasmids and RNAs were transfected by using Lipofectamine 2000 (Invitrogen) according to the manufacturer’s instructions, and cells were harvested or subjected to further treatment at 24 h post-transfection (hpt) or as indicated.

### SG induction, quantification, and drug treatment

For tSG induction, cells were treated with 200 μM AS (Sigma-Aldrich) or incubated at 43°C (HS) for 1 h (or otherwise as indicated) before being harvested for further analysis.

For quantification of the foci of different protein markers in EV71-infected or 2A-expressing cells, EV71- or Myc-tagged cells with Sam68 and TIA-1 foci were counted from cells containing EV71 or Myc tag, and others were counted from cells forming TIA-1 foci. For quantification of tSG formation in EV71-infected or 2A-expressing cells, G3BP was used as an AS- or HS-induced SG marker, and HSP27 was used as an HS-induced SG marker. Cells were considered tSG positive only if they had SGs containing the indicated marker, and the diameter of the biggest SGs in EV71-infected or 2A-expressing cells was at least 1.5 μm (the diameter of the biggest SGs in mock-treated cells was about 3–5 μm).

To distinguish tSGs and aSGs, we treated SG-formed cells with 50 μg/ml CHX (Sigma-Aldrich) for 1 h before fixation. The tSGs would disassemble, and aSGs would remain; as a positive control, cells were treated with AS (200 μM for 0.5 h) to induce tSG formation and then treated with both CHX and AS for another 1 h. In the GuHCl treatments, HeLa or RD cells were infected with 2 mM GuHCl (Sigma-Aldrich) after viral incubation for 1 h; for Western blotting assays, cells were harvested at 6 hpi, and for IF assays, cells were treated with 200 μM AS in the presence of GuHCl for the final 1 h and fixed at 6 hpi.

### Plasmids

The coding regions of G3BP (NCBI accession no. NM_005754.2), TIA-1 (NCBI accession no. NM_022037.2, isoform 1), eIF2α (NCBI accession no. NM_004094.4) and PABP (NCBI accession no. NM_002568.3) were obtained from HeLa cells via RNA extraction and subsequent reverse transcription polymerase chain reaction (RT-PCR) and cloned into the PmeI–Bsp119I sites in pWPI vector with a N-terminal GFP or HA tag (the original region of EMCV and GFP in pWPI was removed). To clone full-length eIF4GI (NCBI accession no. NM_001194947.1, isoform 6), the N-terminal coding region (1–203 aa) was generated by chemosynthesis, and the C-terminal coding region (204–1606 aa) was obtained from HeLa cells via RT-PCR; the full-length clone was generated via overlapping PCR and cloned into pHAGE (puro) vector (substituting fluorescent tag in pHAGE-CMV-MCS-IRES-ZsGreen [EvNO00061605] with puromycin) with an HA tag at the C-terminal. The GFP-PABP^M490P/Q540N^ mutant was generated by using overlapping PCR; PABP^M490P^ was cloned first, and then a Q540N mutation was added.

A plasmid, pCDNA3.0-EV71-CDS, containing completed CDS of EV71 (Hubei-Xiangyang-09, genotype C4) was obtained from Dr. K.L. Wu (State Key Laboratory of Virology, Wuhan University, China). The 5’-UTR was obtained from the total RNAs of EV71-infected HeLa cells by using a 5´ RACE System (Invitrogen) for rapid amplification of cDNA ends according to the manufacturer’s instructions (GSP1, AGGGCAGTGCGTTTATGTATGG; GSP2, GGGTGACTGTCTTCCGTTCCT). The 3’-UTR was obtained from the EV71-infected HeLa cells by using RT-PCR (GGAGAGATCCAGTGGGTTAAG and oligo[dT]_18_). A completed genome of the EV71 clone (pCDNA3.0-EV71) was generated via overlapping PCR and cloned into the pCDNA3.0 vector, and it was taken as a template for creating all other clones that contained the region of the EV71 genome. EV71 proteins were obtained via PCR and cloned into the pCAGGS vector. To generate a vector containing an IRES located in multiple clone sites upstream (pCDNA3.0-IRES), the 5’-UTR of EV71 was cloned into the EcoRI–ClaI sites in the pCDNA3.0 vector. The 2A protease and 2A^C110S^ were obtained via PCR and cloned into the pCDNA3.0-IRES vector. To clone the 2A protease of EV71-BrCr (NCBI accession no. U22521), PV (NCBI accession no. NC_002058.3), and CVA (NCBI accession no. KC117318.1), the coding regions of the three respective viral proteases were generated by chemosynthesis and cloned into the pCDNA3.0-IRES vector.

All the structures were confirmed by DNA sequencing.

### Stable KD cell lines

The shRNA constructs were designed by using the pLKO.1 vector [58] according to protocols recommended by the manufacturer. For stable KD of target protein expression, HEK293T cells were cotransfected with plasmid psPAX2, pMD2.G, and shRNA constructs for 48 h to generate lentiviral particles. The medium was harvested and filtered by a 0.45 μm filter and then divided and stored at -80°C. When the lentivirus was used for infection, HeLa cells were seeded in 6-well plates (1–2 x 10^5^ cells per well), and medium containing the lentivirus was added. After 24 h incubation, the infection medium was replaced with fresh complete growth media. Then, 24 h later, the infected cells were replated and selected in complete growth media with the addition of 2 μg/ml puromycin. After 48 h, the selected cells were replated and selected again in complete growth media with the addition of 2 μg/ml puromycin for another 48 h. Then, the selected cells were maintained in complete growth media plus 1 μg/ml puromycin or subjected to Western blotting to confirm deletion of target proteins. The stable KD cell lines were used for subsequent experiments. The target sequences for the shRNA constructs were: shPKR, GAGGCGAGAAACTAGACAAAG; shNC, GCGCGATAGCGCTAATAATTT.

### Stable overexpression cell lines

Cells were infected with lentiviruses that were generated by cotransfection of plasmid psPAX2, pMD2.G, and target protein expression constructs. The eIF4GI-HA-HeLa and eIF4GI^G689E^-HA-HeLa cell lines were obtained as previously described for stable KD cell lines. For the other cell lines with overexpression of GFP-tagged targets, GFP-positive cells were sorted from the infected cells via flow cytometry and cultured in complete growth media. The cell lines with stable overexpression were used for subsequent experiments.

### Construction and generation of recombinant viruses

The full-length recombinant EV71 infectious clone was constructed into a pBS vector bearing a T7 promoter upstream of the virus genome (pBS-T7-EV71). The 2A protease activity-deficient EV71 infectious clone was constructed using site-directed mutagenesis via overlapping PCR (pBS-T7-EV71-2A^C110S^). An IRES structure was inserted between the sequence of VP1 and 2A to counteract the 2A^pro^ defect. Constructs were then linearized by restriction enzyme and purified by phenol:chloroform and ethanol precipitation. To package EV71 viruses, viral RNA was transcribed using the TranscriptAid T7 High-Yield Transcription Kit (ThermoFisher Scientific) and purified using the RNeasy Mini Kit (Qiagen). The viral RNA (2 μg) transcripts were transfected into HeLa cells grown in a monolayer on 6-well plates by using Lipofectamine 2000 for 3 days. The supernatants were passaged on fresh RD cells for further amplification. After 3 days, the supernatants were collected and stored at -80°C, and the cells were collected and divided into two groups, one for Western blotting to confirm the viral infection and the other for RT-PCR and DNA sequencing to confirm the mutation. A single recovered recombinant EV71-2A^C110S^ was isolated by removing the agar plug during a plaque assay. The agar plug was dissolved in 500 μl of opti-MEM overnight at 4°C, and half was used for EV71-2A^C110S^ amplification by infecting RD cell monolayers. Finally, the whole genome sequence of EV71-2A^C110S^ was confirmed by RT-PCR and DNA-sequencing, and the titer of EV71-2A^C110S^ was measured via plaque assay.

### IF and western blot assays

For IF, cells were fixed with 4% (wt/vol) paraformaldehyde/phosphate-buffered saline (PBS) and permeated with 0.2% (wt/vol) Triton X-100/PBS solution at room temperature (RT) for 20 min, respectively, and then blocked with 3% (wt/vol) bovine serum albumin (BSA) in PBS at RT for 30 min. Primary antibodies were diluted in 1% (wt/vol) BSA/PBS and incubated overnight at 4°C, followed by incubation of secondary antibodies at RT for 2 h. The following dye-conjugated secondary antibodies were used for this analysis: Alexa Fluor 647 donkey anti-goat immunoglobulin (IgG) H+L, Alexa Fluor 488 donkey anti-rabbit IgG H+L, and Alexa Fluor 594 donkey anti-mouse IgG H+L (Life Technologies). After being stained with 1 μg/ml DAPI (Roche) in PBS for 5 min, cells were mounted with Prolong Diamond Antifade Mountant (Life Technology) and examined on a Leica confocal microscope.

For Western blotting, cells were harvested and lysed in lysis buffer (150 nM NaCl, 50 nM Tris-HCl [pH 7.4],1% Triton X-100, 1 mM EDTA [pH 8.0], and 0.1% sodium dodecyl sulfate [SDS]) with a protease inhibitor cocktail, incubated on ice for 30 min, and centrifuged at 4°C for 30 min at 12,000 g. The supernatants were boiled in SDS-polyacrylamide gel electrophoresis (PAGE) loading buffer at 100°C for 10 min and then resolved on SDS-PAGE and detected on a Fujifilm LAS-4000 imaging system. The indicated primary and horseradish peroxidase-conjugated secondary antibodies (ThermoFisher Scientific) were used.

The following primary antibodies were used: mouse monoclonal anti-c-Myc (Cat #sc-40), rabbit polyclonal anti-c-Myc (Cat #sc-789), rabbit monoclonal anti-Sam68 (Cat #sc-333), goat polyclonal anti-TIA-1 (Cat #sc-1751), mouse monoclonal anti-GAPDH (Cat #sc-32233) and rabbit monoclonal anti-GFP (Cat #sc-8334) were purchased from Santa Cruz Biotechnology. Mouse monoclonal anti-G3BP (Cat #611127) and mouse monoclonal anti-TIAR (Cat #610352) were purchased from BD Transduction Laboratories. Mouse monoclonal anti-β-actin (Cat #AC004), rabbit polyclonal anti-eIF4A (Cat #A5294), rabbit polyclonal anti-eIF4E (Cat #A2162), rabbit polyclonal anti-eIF1a (Cat #A5917), rabbit polyclonal anti-eIF3a (Cat #A0573), rabbit polyclonal anti-RPS3 (Cat #A11131), and rabbit polyclonal anti-3C (Cat #A10003) were purchased from ABclonal. Rabbit monoclonal anti-eIF4G (Cat #2469S), rabbit monoclonal anti-p-eIF2α (Cat #9721), and rabbit monoclonal anti-eIF2α (Cat #9722) were purchased from Cell Signaling Technology. Mouse monoclonal anti-Flag (Cat #F1804), mouse monoclonal anti-HA (Cat #H9658), and rabbit monoclonal anti-HA (Cat #H6908) were purchased from Sigma-Aldrich. Mouse monoclonal anti-PABP (Cat #ab6125) was purchased from Abcam. Mouse monoclonal anti-HSP27 (Cat #ADI-SPA-800D) was purchased from StressGen. Mouse monoclonal anti-EV71 (Cat #MAB979) and Mouse monoclonal anti-puromycin, clone 12D10 (Cat #MABE343) were purchased from Millipore. Mouse monoclonal anti-VP1 was purchased from Abmax (Clone 22A14) [59].

### FISH

For detection of total polyadenylated mRNA (polyA+ mRNA), cells were plated on coverslips and incubated overnight before treatment with AS, EV71, or 2A. Cells were fixed with 2% formaldehyde for 10 min and processed as previously described [60] by using a 3’-biotinylated oligo(dT)_40_ probe. Cells were then processed as those described for the aforementioned IF assays. The oligo(dT)_40_ probe was visualized by streptavidin conjugated to cyanin 3 (Cy3).

The ViewRNA ISH Cell Assay Kit and probes for EV71 positive-strand RNA (+vRNA) and PABPC1 mRNA were purchased from Affymetrix and used to detect target mRNAs according to protocols recommended by the manufacturer.

### Luciferase reporter assays

A Renilla luciferase gene sequence was constructed into the aforementioned EV71 infectious clone backbone (pBS-T7-EV71) by replacing the whole viral protein-coding sequence (UTR^EV71^-Rluc). The UTR^PABPC1^-Rluc reporter was generated by replacing the EV71 UTR region by PABPC1 UTR region. The RNAs were transcribed using the TranscriptAid T7 High-Yield Transcription Kit (ThermoFisher Scientific) and purified using the RNeasy Mini Kit (Qiagen). To evaluate the influence of aSGs on EV71 or PABPC1 translation, eIF4GI-HA- and eIF4GI^G689E^-HA-HeLa cells were seeded in 24-well plates, and the RNA (0.4 μg/well) transcripts were transfected into the cells after EV71 (MOI = 10) infection for 3 hours or EV71-2A^C110S^ (MOI = 10) infection for 6 hours, then analyzed the reporter expression at 1.5 hpt and 3 hpt. Cells infected with EV71-2A^C110S^ served as negative control and analyzed the reporter expression at 3 hpt. To evaluate the influence of tSGs on EV71 or PABPC1 translation, shNC- and shPKR-HeLa cells were seeded in 24-well plates and the RNA (0.4 μg/well) transcripts were transfected into the cells after EV71-2A^C110S^ (MOI = 10) infection for 6 h or EV71 (MOI = 10) infection for 3 h. The reporter expression in EV71-2A^C110S^-infected cells were analyzed at 3 hpt and 6 hpt and the reporter expression in EV71-infected cells were analyzed at 3 hpt as control. Renilla luciferase activity was assessed by using a Renilla Luciferase Assay Kit (Promega) according to the manufacturer’s instructions. All experiments were performed in triplicate, and assays were repeated at least three times.

### Statistical analysis

Statistical analysis was performed using GraphPad Prism v6.01. All results are expressed as means ± SD of at least three independent experiments (n≥3). The p value was calculated using an unpaired Student's t-test. In all tests, p>0.05 was considered non-statistically significant (n.s.), and p<0.05 was considered statistically significant, marked as follows: *, p<0.05; **, p<0.01; ***, p<0.001.

## Supporting information

S1 Fig Localization of SG components during EV71 infection. (A-G) HeLa cells were mock-infected or infected with EV71 (MOI = 10) for consecutive times (2, 4, and 6 h; mock-infected cells were converged at 2 h) and then stained with antibodies to visualize the protein foci. DAPI (blue) was used to stain the nuclei. Arrows indicate the relative localization of the proteins to the TIA-1 foci.
(H) Quantitative analysis of HeLa (left panel) or RD (right panel) cells with EV71 fluorescence or with foci of the indicated proteins (infected as in A). n = 3, 300 cells/condition were counted, mean±SD.
Scale bars, 10 μm.
(TIF)

S2 Fig 2A induces the formation of tSGs and of p-bodies in EV71-infected or 2A-expressing cells. (A)Assessment of the ability of EV71 proteins to induce TIA-1 foci. HeLa or RD cells were transfected with Myc-tagged viral proteins for 24 h and then fixed and stained with antibodies against Myc (red), TIA-1 (green), and DAPI (blue). For RD cells, only 2A-transfected cells are shown (bottom and left panel). Arrows indicate the cells with TIA-1 foci.
(B)HeLa cells were transiently transfected with Myc-tagged 2A for 24 h and then stained with antibodies as indicated. Anti-Myc antibody showed the expression of 2A, and DAPI (blue) was used to stain the nuclei. Arrows indicate the relative localization of the proteins to the TIA-1 foci. Quantitative analysis of the cells with foci of the indicated proteins (bottom and right panel). Each marker protein was evaluated among 2A-expressing cells individually. n = 3, 240 cells/condition were counted, mean±SD; ***p<0.001.
(C)Formation of p-bodies in EV71-infected cells. HeLa cells were infected with EV71 (MOI = 10) or treated as indicated. Anti-DCP1A antibody was used to visualize p-bodies, anti-TIA-1 antibody was used to visualize EV71-induced aSGs, and DAPI (blue) was used to stain the nuclei.
(D)Quantitative analysis of the cells in C with p-bodies. n = 3, 300 cells/condition were counted, mean±SD; n.s., no statistical significance, ***p<0.001.
Scale bars, 10 μm.
(TIF)

S3 Fig The cleavage of PABP has no influence on aSG formation. (A-C) HeLa cells were transfected with GFP-tagged PABP or PABP^M470P/Q540N^ for 24 h, followed by 2A transfection. The cleavage of PABP and PABP^M470P/Q540N^ was analyzed by WB (A). The formation of aSGs in PABP/PABP^M470P/Q540N^-expressing cells was viewed by IF assay, and anti-TIA-1 antibody was used to visualize 2A-induced aSGs. Scale bars, 10 μm (B). Quantitative analysis of the PABP/PABP^M470P/Q540N^-expressing cells with TIA-1-marked aSGs in B. n = 3, 240 cells/condition were counted, mean±SD; n.s., no statistical significance (C).
(TIF)

S4 Fig EV71 blocks tSG formation independent of eIF2α phosphorylation and 3C. (A and B) RD cells were treated as in Fig 3A and quantified as in Fig 3B.
(C) The level of eIF2α phosphorylation after AS or HS treatment in EV71-infected HeLa/RD cells. Cells were treated as in Fig 3A and subjected to WB. Arrows indicates eIF4G cleavage products.
(D) Confirmation of GFP-G3BP^Q326E^ resistance to cleavage by 3C (left panel) and analysis of the effect of GFP-G3BP^Q326E^ and 3C on AS-induced SG formation (right panel) in HeLa cells. Arrow indicates GFP-G3BP cleavage products.
(E) Analysis of the effects of GFP-G3BP^Q326E^ on tSG formation in EV71-infected cells. GFP-G3BP- or GFP-G3BP^Q326E^-RD cells were treated and stained as in Fig 3D. Shown is tSG formation at 4 hpi and 6 hpi (left panel). Quantitative analysis of EV71-infected cells with tSGs in left panel. n = 3, 300 cells/condition were counted, mean±SD; ***p<0.001 (right panel).
(F) GuHCl effects on tSG formation in EV71-infected RD cells. RD cells were treated and analyzed as in Fig 3F (top and left panel), 3G (bottom panel), and 3H (top and right pamel).
“+” in D indicates the 3C-expressing cells, “+” in others indicates the infected cells, and yellow arrows indicate the uninfected cells. Scale bars, 10 μm.
(TIF)

S5 Fig 2A protease can block tSG formation. (A and B) IF of RD cells treated as in Fig 4A and 4B (A) and quantified as in Fig 4C (B).
(C) HeLa and RD cells were treated as in Fig 4A and 4B and harvested at 12 and 24 hpt for WB to detect the phosphorylation of eIF2α and the cleavage of eIF4G. Arrows indicate eIF4G cleavage products.
(D) GFP-G3BP^Q326E^-HeLa cells were transfected with Myc-tagged 2A for 24 h, followed by treatment with AS for another 1 h. Cells were stained with Myc (magenta) and TIA-1 (red), and GFP-G3BP^Q326E^ (green) served as a marker of tSGs.
(E) Quantitative analysis of 3C- (in S4D Fig) or 2A-expressing cells with tSGs in D. n = 3, 240 cells/condition were counted, mean±SD; ***p<0.001.
(F and G) The effects of eIF4GI^G689E^ on tSG formation in 2A-expressing cells. The eIF4GI-HA- and eIF4GI^G689E^-HA-HeLa cells were transfected with 2A for 24h, followed by treatment with AS for another 1 h. Cells were stained with eIF4A (green) and G3BP (red) to visualize tSGs, and DAPI (blue) was used to stain the nuclei (F). Quantitative analysis of cells with tSGs in F. n = 3, 240 cells/condition were counted, mean±SD; n.s., no statistical significance (G).
“+” indicates the 2A-expressing cells, and yellow arrows indicate the cells without 2A expression. Scale bars, 10 μm.
(TIF)

S6 Fig The characteristics of EV71-2A^C110S^ and the effect of EV71-2A^C110S^ on HS-induced SG formation. (A)Replication kinetics (top panel) and VP1 expression (bottom panel) of EV71 and EV71-2A^C110S^ in HeLa cells (MOI = 0.5). β-actin was the sample loading control. “*” indicated decrease of β-actin which induced by lytic infection of EV71. The experiment was repeated three times.
(B)The effect of EV71- and EV71-2A^C110S^ infection on host cellular translation. HeLa cells were infected with EV71-2A^C110S^ (MOI = 5) or EV71 (MOI = 1 and MOI = 5) for consecutive times and treated with puromycin (10μg/ml) for 30 min prior to harvest. Controls are cells mock-infected and treated with puromycin or without puromycin. Cell lysates were analyzed via WB with antibodies against puromycin, VP1 and β-actin. β-actin was the sample loading control.
(C)HeLa cells were infected with EV71 or EV71-2A^C110S^ as in Fig 5A and then treated with HS for 1 h before fixation. Cells were then stained with 3C (magenta), TIA-1 (green), or HSP27 (red). “+” indicates the infected cells, and yellow arrows indicate the uninfected cells. Scale bars, 10 μm.
(D)Quantitative analysis of infected cells with tSGs (HSP27) in C. n = 3, 300 cells/condition were counted, mean±SD; ***p<0.001.
(TIF)

S7 Fig Effects of EV71-induced aSGs and EV71^C110S^-induced tSGs on PABPC1 mRNA translation. (A-C) Graphic description of PABPC1-UTR driven Renilla luciferase reporter (UTR^PABPC1^-Rluc) expression (A) and analysis of translation efficiency of UTR^PABPC1^-Rluc mRNA as described in Fig 7B (B) and Fig 7C (C). n = 3, mean±SD; n.s., no statistical significance; *p<0.05; **p<0.01; ***p<0.001.
(TIF)

S8 Fig 2A-induced aSG formation by cleaving eIF4GI is common among picornaviruses. (A-C) The eIF4GI-HA- and eIF4GI^G689E^-HA-HeLa cells were transfected with 2A of EV71-BrCr, CVA or PV for 24h. Cleavage of eIF4GI-HA and eIF4GI^G689E^-HA were analyzed via WB assay (A). Cells were stained with Sam68 and G3BP and analyzed via IF assay. “+” indicates the 2A-expressing cells, and yellow arrows indicate the cells without 2A expression. Scale bars, 10 μm (B). Quantitation of eIF4GI-HA- and eIF4GI^G689E^-HA-HeLa cells with aSGs in the presence of 2A of EV71-BrCr, CVA, and PV. n = 3, 240 cells/condition were counted, mean±SD; ***p<0.001.
(TIF)
