# Supplementary figures and images for: Picornavirus 2A protease regulates stress granule formation to facilitate viral translation
Source: PLoS Pathog. 2018 Feb 7;14(2):e1006901. doi: 10.1371/journal.ppat.1006901 (PMC5819834; doi:10.1371/journal.ppat.1006901)

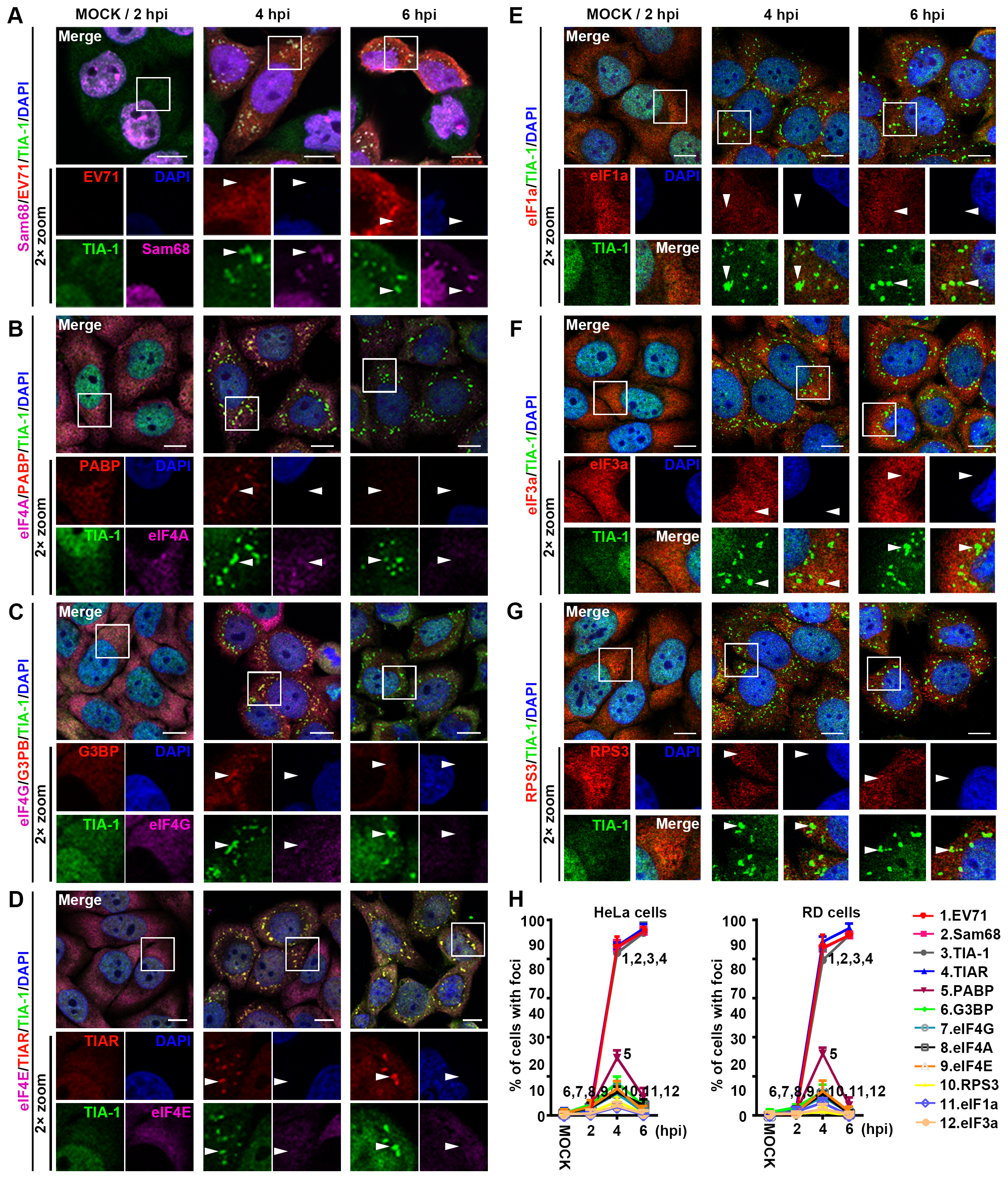

Supplement: S1 Fig — (A-G) HeLa cells were mock-infected or infected with EV71 (MOI = 10) for consecutive times (2, 4, and 6 h; mock-infected cells were converged at 2 h) and then stained with antibodies to visualize the protein foci. DAPI (blue) was used to stain the nuclei. Arrows indicate the relative localization of the proteins to the TIA-1 foci. (H) Quantitative analysis of HeLa (left panel) or RD (right panel) cells with EV71 fluorescence or with foci of the indicated proteins (infected as in A). n = 3, 300 cells/condition were counted, mean±SD. Scale bars, 10 μm. (TIF) [file ppat.1006901.s001.tif]

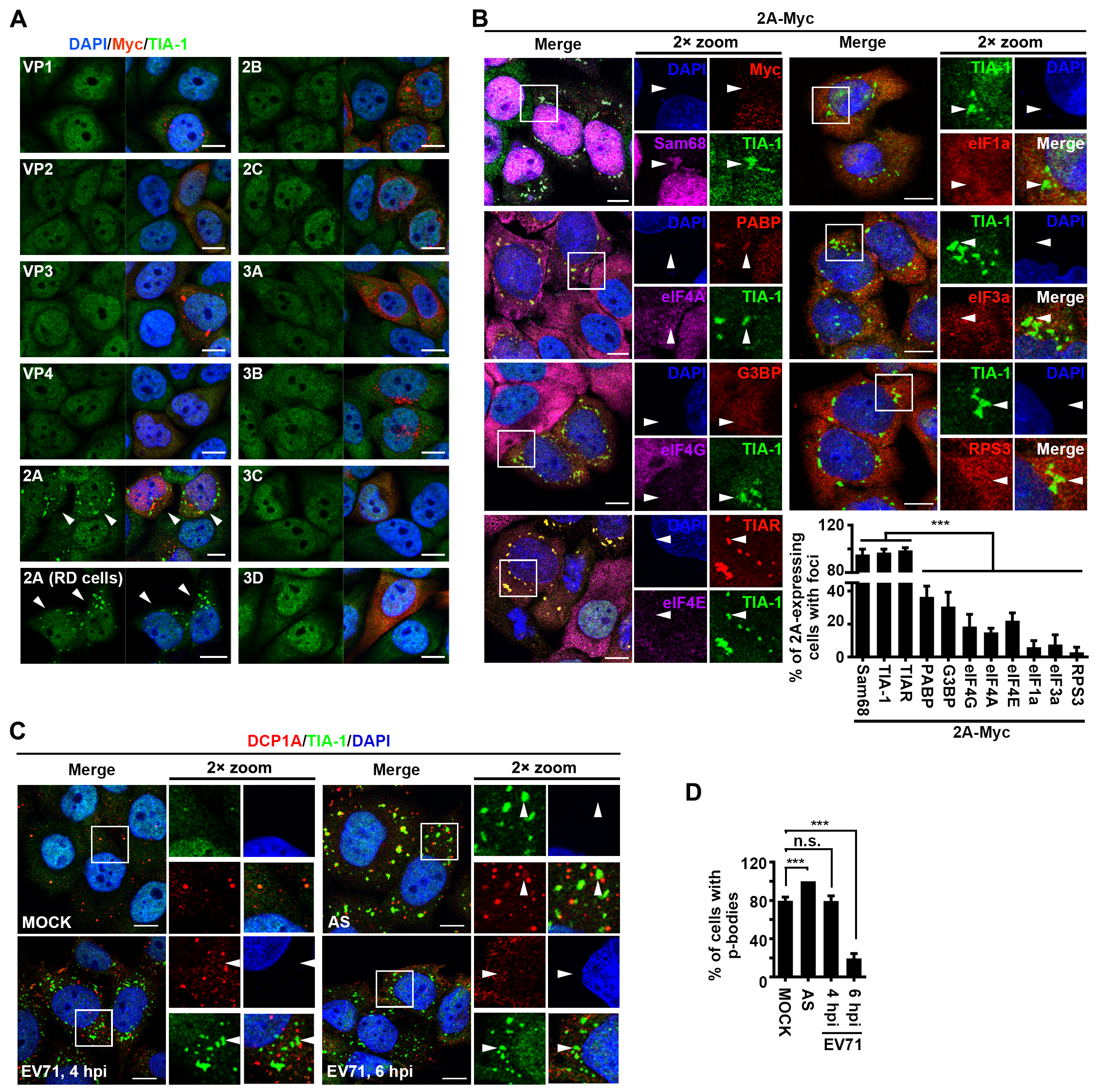

Supplement: S2 Fig — (A)Assessment of the ability of EV71 proteins to induce TIA-1 foci. HeLa or RD cells were transfected with Myc-tagged viral proteins for 24 h and then fixed and stained with antibodies against Myc (red), TIA-1 (green), and DAPI (blue). For RD cells, only 2A-transfected cells are shown (bottom and left panel). Arrows indicate the cells with TIA-1 foci. (B)HeLa cells were transiently transfected with Myc-tagged 2A for 24 h and then stained with antibodies as indicated. Anti-Myc antibody showed the expression of 2A, and DAPI (blue) was used to stain the nuclei. Arrows indicate the relative localization of the proteins to the TIA-1 foci. Quantitative analysis of the cells with foci of the indicated proteins (bottom and right panel). Each marker protein was evaluated among 2A-expressing cells individually. n = 3, 240 cells/condition were counted, mean±SD; ***p<0.001. (C)Formation of p-bodies in EV71-infected cells. HeLa cells were infected with EV71 (MOI = 10) or treated as indicated. Anti-DCP1A antibody was used to visualize p-bodies, anti-TIA-1 antibody was used to visualize EV71-induced aSGs, and DAPI (blue) was used to stain the nuclei. (D)Quantitative analysis of the cells in C with p-bodies. n = 3, 300 cells/condition were counted, mean±SD; n.s., no statistical significance, ***p<0.001. Scale bars, 10 μm. (TIF) [file ppat.1006901.s002.tif]

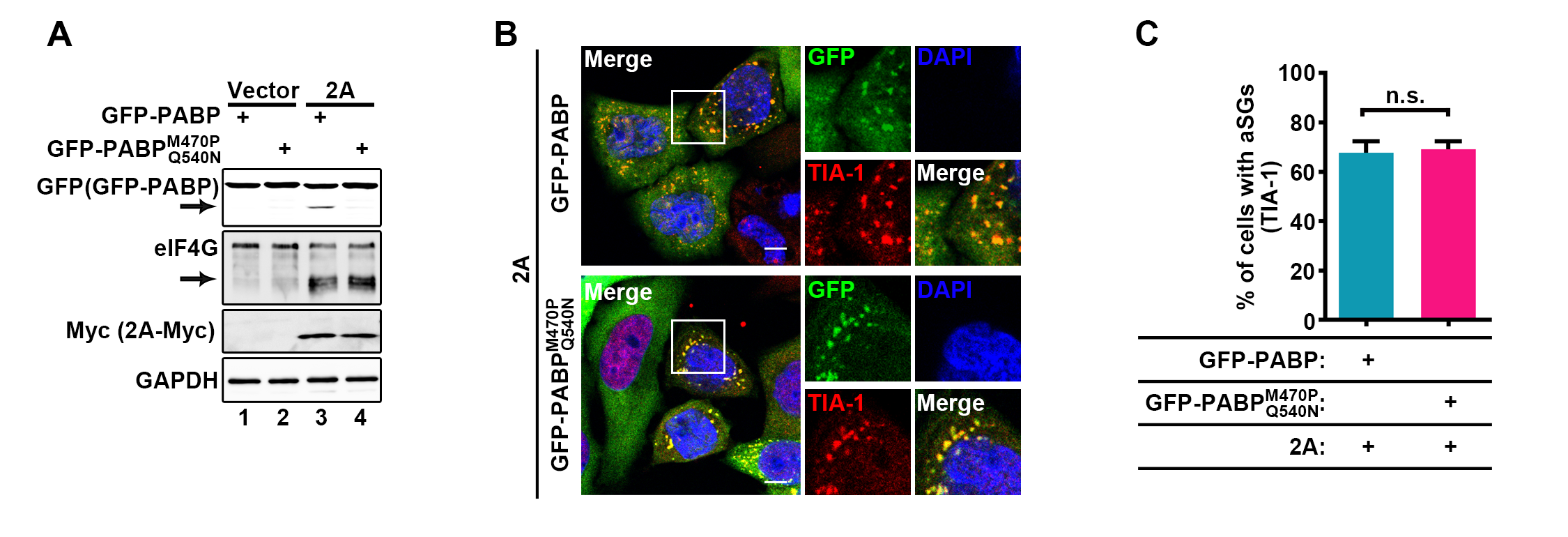

Supplement: S3 Fig — (A-C) HeLa cells were transfected with GFP-tagged PABP or PABPM470P/Q540N for 24 h, followed by 2A transfection. The cleavage of PABP and PABPM470P/Q540N was analyzed by WB (A). The formation of aSGs in PABP/PABPM470P/Q540N-expressing cells was viewed by IF assay, and anti-TIA-1 antibody was used to visualize 2A-induced aSGs. Scale bars, 10 μm (B). Quantitative analysis of the PABP/PABPM470P/Q540N-expressing cells with TIA-1-marked aSGs in B. n = 3, 240 cells/condition were counted, mean±SD; n.s., no statistical significance (C). (TIF) [file ppat.1006901.s003.tif]

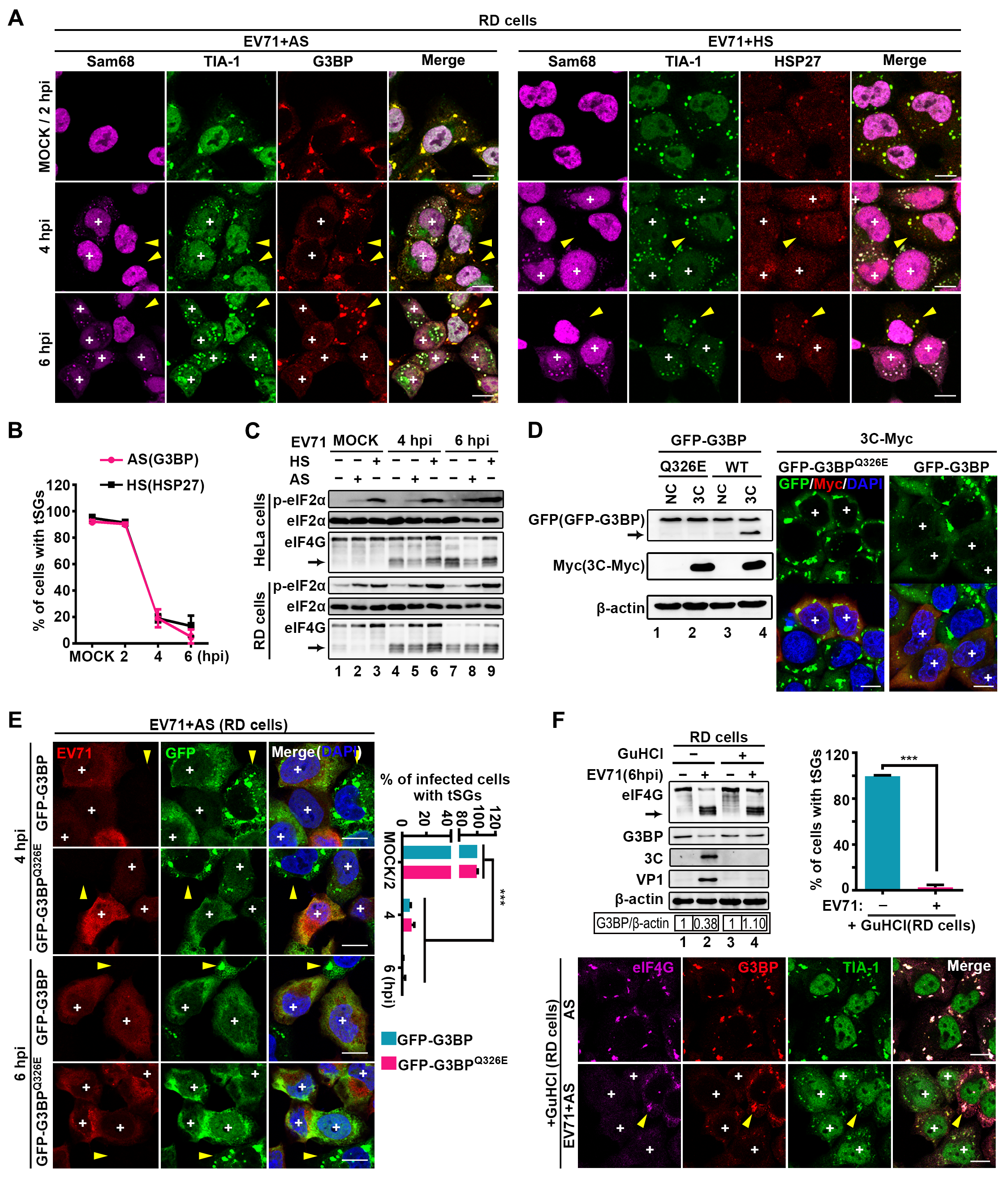

Supplement: S4 Fig — (A and B) RD cells were treated as in Fig 3A and quantified as in Fig 3B. (C) The level of eIF2α phosphorylation after AS or HS treatment in EV71-infected HeLa/RD cells. Cells were treated as in Fig 3A and subjected to WB. Arrows indicates eIF4G cleavage products. (D) Confirmation of GFP-G3BPQ326E resistance to cleavage by 3C (left panel) and analysis of the effect of GFP-G3BPQ326E and 3C on AS-induced SG formation (right panel) in HeLa cells. Arrow indicates GFP-G3BP cleavage products. (E) Analysis of the effects of GFP-G3BPQ326E on tSG formation in EV71-infected cells. GFP-G3BP- or GFP-G3BPQ326E-RD cells were treated and stained as in Fig 3D. Shown is tSG formation at 4 hpi and 6 hpi (left panel). Quantitative analysis of EV71-infected cells with tSGs in left panel. n = 3, 300 cells/condition were counted, mean±SD; ***p<0.001 (right panel). (F) GuHCl effects on tSG formation in EV71-infected RD cells. RD cells were treated and analyzed as in Fig 3F (top and left panel), 3G (bottom panel), and 3H (top and right pamel). “+” in D indicates the 3C-expressing cells, “+” in others indicates the infected cells, and yellow arrows indicate the uninfected cells. Scale bars, 10 μm. (TIF) [file ppat.1006901.s004.tif]

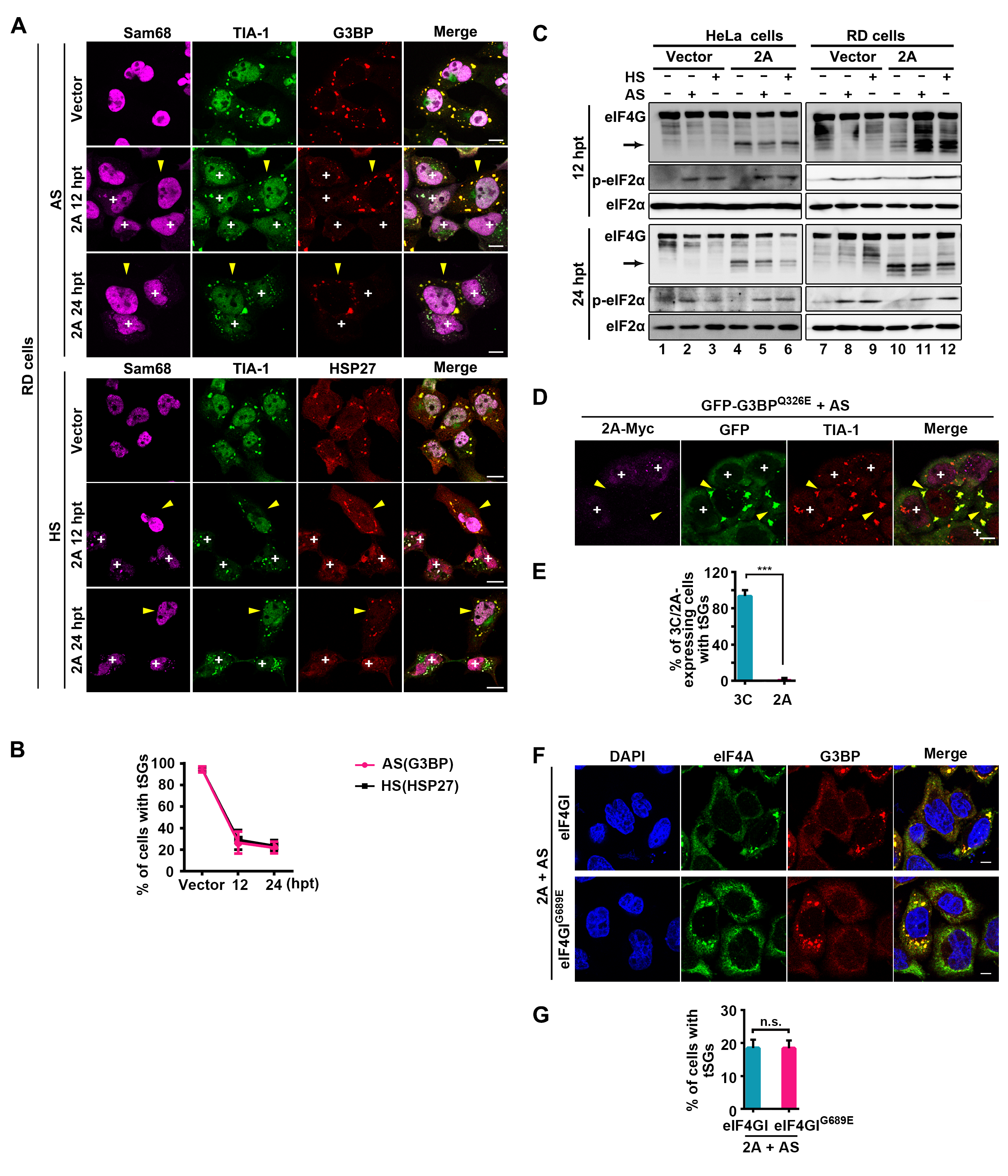

Supplement: S5 Fig — (A and B) IF of RD cells treated as in Fig 4A and 4B (A) and quantified as in Fig 4C (B). (C) HeLa and RD cells were treated as in Fig 4A and 4B and harvested at 12 and 24 hpt for WB to detect the phosphorylation of eIF2α and the cleavage of eIF4G. Arrows indicate eIF4G cleavage products. (D) GFP-G3BPQ326E-HeLa cells were transfected with Myc-tagged 2A for 24 h, followed by treatment with AS for another 1 h. Cells were stained with Myc (magenta) and TIA-1 (red), and GFP-G3BPQ326E (green) served as a marker of tSGs. (E) Quantitative analysis of 3C- (in S4D Fig) or 2A-expressing cells with tSGs in D. n = 3, 240 cells/condition were counted, mean±SD; ***p<0.001. (F and G) The effects of eIF4GIG689E on tSG formation in 2A-expressing cells. The eIF4GI-HA- and eIF4GIG689E-HA-HeLa cells were transfected with 2A for 24h, followed by treatment with AS for another 1 h. Cells were stained with eIF4A (green) and G3BP (red) to visualize tSGs, and DAPI (blue) was used to stain the nuclei (F). Quantitative analysis of cells with tSGs in F. n = 3, 240 cells/condition were counted, mean±SD; n.s., no statistical significance (G). “+” indicates the 2A-expressing cells, and yellow arrows indicate the cells without 2A expression. Scale bars, 10 μm. (TIF) [file ppat.1006901.s005.tif]

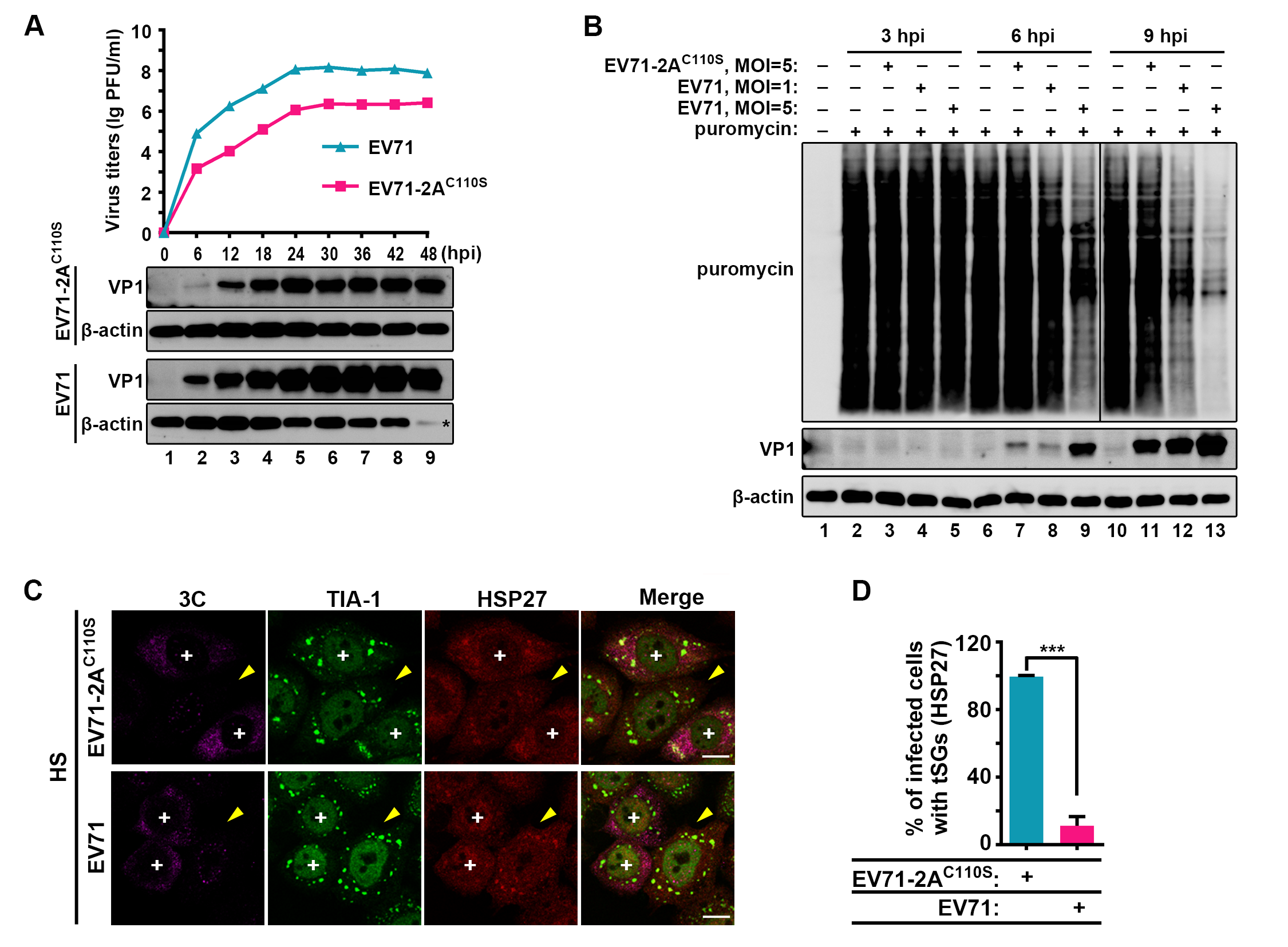

Supplement: S6 Fig — (A)Replication kinetics (top panel) and VP1 expression (bottom panel) of EV71 and EV71-2AC110S in HeLa cells (MOI = 0.5). β-actin was the sample loading control. “*” indicated decrease of β-actin which induced by lytic infection of EV71. The experiment was repeated three times. (B)The effect of EV71- and EV71-2AC110S infection on host cellular translation. HeLa cells were infected with EV71-2AC110S (MOI = 5) or EV71 (MOI = 1 and MOI = 5) for consecutive times and treated with puromycin (10μg/ml) for 30 min prior to harvest. Controls are cells mock-infected and treated with puromycin or without puromycin. Cell lysates were analyzed via WB with antibodies against puromycin, VP1 and β-actin. β-actin was the sample loading control. (C)HeLa cells were infected with EV71 or EV71-2AC110S as in Fig 5A and then treated with HS for 1 h before fixation. Cells were then stained with 3C (magenta), TIA-1 (green), or HSP27 (red). “+” indicates the infected cells, and yellow arrows indicate the uninfected cells. Scale bars, 10 μm. (D)Quantitative analysis of infected cells with tSGs (HSP27) in C. n = 3, 300 cells/condition were counted, mean±SD; ***p<0.001. (TIF) [file ppat.1006901.s006.tif]

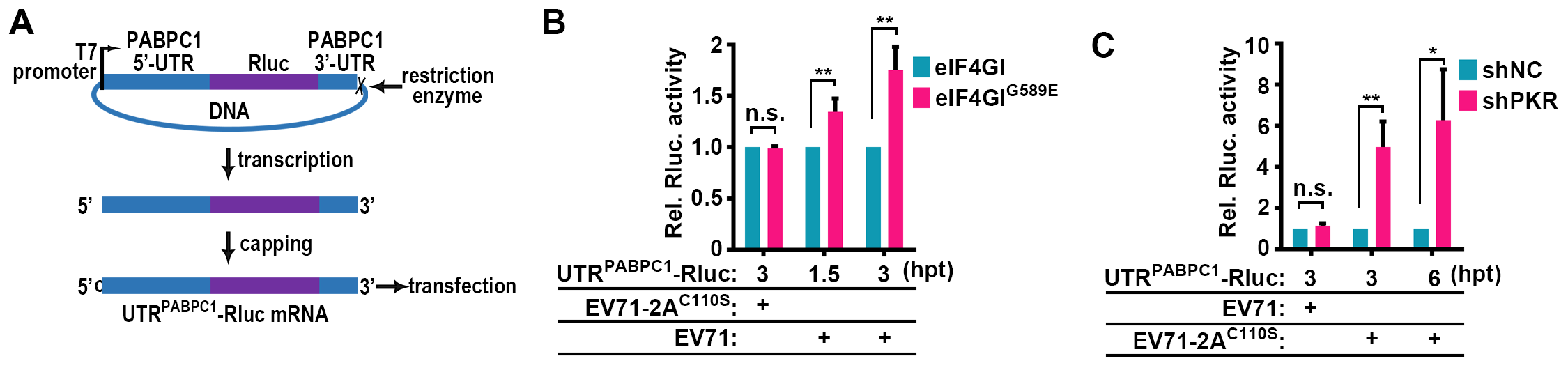

Supplement: S7 Fig — (A-C) Graphic description of PABPC1-UTR driven Renilla luciferase reporter (UTRPABPC1-Rluc) expression (A) and analysis of translation efficiency of UTRPABPC1-Rluc mRNA as described in Fig 7B (B) and Fig 7C (C). n = 3, mean±SD; n.s., no statistical significance; *p<0.05; **p<0.01; ***p<0.001. (TIF) [file ppat.1006901.s007.tif]

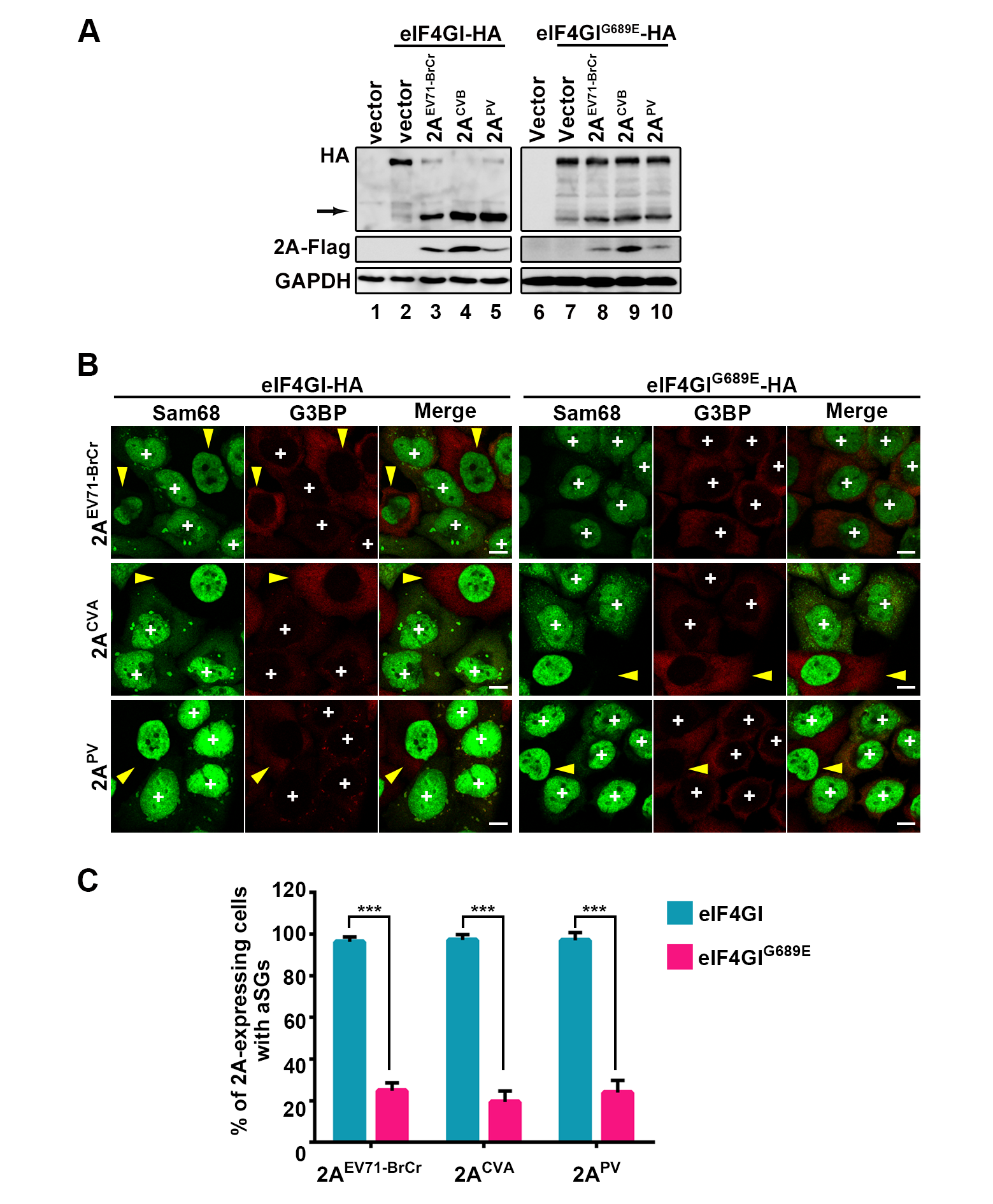

Supplement: S8 Fig — (A-C) The eIF4GI-HA- and eIF4GIG689E-HA-HeLa cells were transfected with 2A of EV71-BrCr, CVA or PV for 24h. Cleavage of eIF4GI-HA and eIF4GIG689E-HA were analyzed via WB assay (A). Cells were stained with Sam68 and G3BP and analyzed via IF assay. “+” indicates the 2A-expressing cells, and yellow arrows indicate the cells without 2A expression. Scale bars, 10 μm (B). Quantitation of eIF4GI-HA- and eIF4GIG689E-HA-HeLa cells with aSGs in the presence of 2A of EV71-BrCr, CVA, and PV. n = 3, 240 cells/condition were counted, mean±SD; ***p<0.001. (TIF) [file ppat.1006901.s008.tif]
